# Exercise patterns and atherosclerosis: a network meta-analysis based on randomized controlled trials

**DOI:** 10.3389/fphys.2026.1911464

**Published:** 2026-08-18

**Authors:** Cheng Xu, Shuxin Yan, Yuxin Liu, Hui Zhang, Fei Li, Yu Zhang, Yiying Liu, Yulian Yuan, Shujing Zhang, Xianggen Zhong, Yan Sun

**Affiliations:** 1 School of Chinese Medicine, Beijing University of Chinese Medicine, Beijing, China; 2 Guang’anmen Hospital, China Academy of Chinese Medical Sciences, Beijing, China; 3 Tsinghua University Yuquan Hospital, Tsinghua University, Beijing, China

**Keywords:** arterial stiffness, atherosclerotic cardiovascular disease, exercise prescription, lipid profile, network meta-analysis

## Abstract

**Background:**

In 2023, cardiovascular diseases caused an estimated 19.2 million deaths worldwide, accounting for approximately one-third of all global deaths. Despite this heavy burden, there is limited evidence comparing the effects of various exercise modalities on functional capacity, arterial stiffness, and lipid profiles in patients with atherosclerotic cardiovascular disease (ASCVD). This network meta-analysis evaluated 12 exercise interventions to guide personalized exercise prescriptions.

**Methods:**

A systematic search of PubMed, Embase, the Cochrane Library, and Web of Science was conducted from their inception until June 10, 2026, to identify randomized controlled trials assessing exercise interventions in adults with ASCVD. The primary outcomes measured included the 6-minute walk distance (6MWD), pulse wave velocity (PWV), total cholesterol (TC), triglycerides (TG), high-density lipoprotein cholesterol (HDL-C), and low-density lipoprotein cholesterol (LDL-C). A network meta-analysis was conducted, and the ranking of interventions was determined using the surface under the cumulative ranking curve (SUCRA). The protocol was registered with PROSPERO (registration No. CRD420261412964).

**Results:**

A comprehensive analysis incorporating 29 RCTs with a cumulative sample size of 2,496 participants was conducted. The network meta-analysis (NMA) indicated that various exercise modalities exhibited unique benefits for specific health outcomes. Resistance Training (RT) was identified as the most effective intervention for enhancing the 6MWD. Both Qigong combined with Dancing (QD) and Aerobic Exercise (AE) were associated with the most pronounced improvements in PWV. In terms of lipid profile modulation, QD was the most efficacious in reducing total cholesterol levels and increasing HDL-C, whereas RT combined with Blood Flow Restriction (BFRT) was superior in improving LDL-C levels.

**Conclusion:**

Different exercise interventions yield specific benefits in managing ASCVD. This study endorses the concept of “goal-oriented exercise prescription” and suggests that exercise regimens should be meticulously tailored to align with patients’ individualized treatment goals, in conjunction with standard pharmacological therapy.

**Systematic Review Registration:**

https://www.crd.york.ac.uk/PROSPERO/view/CRD420261412964, identifier CRD420261412964.

## 1 Introduction

Among the four predominant chronic diseases, atherosclerotic cardiovascular disease (ASCVD) encompasses a spectrum of clinical cardiovascular conditions resulting from the development and progression of atherosclerotic plaques within arterial walls. This disease primarily includes acute coronary syndrome, previous myocardial infarction, stable or unstable angina, coronary or other arterial revascularization, ischemic stroke, transient ischemic attack, and peripheral artery disease (Grundy et al., 2019). ASCVD is characterized by a high global incidence and mortality rate and represents the leading cause of vascular occlusion at various levels, as well as myocardial and cerebral infarctions. The progression and manifestation of ASCVD are influenced by factors such as gender, age, obesity, and lifestyle habits (Doi et al., 2023), and it exerts a substantial impact on the physical and mental health of affected individuals (Toval et al., 2025). Consequently, the development and implementation of health intervention strategies that address both the physical and psychological challenges faced by ASCVD patients are essential for enhancing their quality of life (Makover et al., 2022).

The pathogenesis of ASCVD is intricately associated with inflammatory responses and lipid deposition. Consequently, contemporary management and treatment strategies predominantly target these two pathological processes (Bäck et al., 2019; Zhong et al., 2019). Therapeutic interventions primarily involve the administration of lipid-lowering agents, supplemented by antiplatelet and antihypertensive medications, including statins, cholesterol absorption inhibitors, Proprotein convertase subtilisin/kexin type 9 inhibitors, and aspirin. These interventions aim to alleviate arterial blockages, mitigate the risk of myocardial and cerebrovascular infarctions, and relieve various painful complications (Ali et al., 2021; Arnold et al., 2021; Henein et al., 2022; Passacquale et al., 2022; Gallo et al., 2025). Nonetheless, for patients who are either intolerant to statins or have developed resistance, non-pharmacological interventions, such as exercise therapy, play a pivotal role in enhancing the quality of life and functional capacity of individuals with ASCVD. These therapies, to some extent, synergistically augment the effects of pharmacological treatments, decelerate disease progression, and further improve the quality of life (Waksman et al., 2024).

Prior research has predominantly concentrated on distinct cardiovascular cohorts or a narrow spectrum of exercise-related outcomes. For instance, a recent network meta-analysis assessed the quality of life and mental health in individuals with coronary artery disease (Toval et al., 2025). However, there is a paucity of comparative evidence concerning functional capacity, arterial stiffness, and lipid profiles within the broader ASCVD population. Furthermore, the differential impacts of various exercise modalities on these clinically significant outcomes remain ambiguous due to the scarcity of direct head-to-head comparisons. This evidentiary gap poses challenges for clinicians in selecting exercise interventions tailored to specific therapeutic objectives, such as enhancing functional capacity, diminishing arterial stiffness, or modulating lipid profiles.

This study adhered to established guidelines for conducting a network meta-analysis (NMA) based on randomized controlled trials (RCTs). Network meta-analysis is a sophisticated meta-analytic technique that integrates both direct and indirect comparative evidence, facilitating the comparison of the relative effects of multiple interventions within a single evidence framework. This approach addresses the limitations inherent in traditional pairwise meta-analyses, which are restricted to pairwise comparisons. The principal advantage of NMA lies in its capacity to construct an evidence network using common controls, thereby enabling a comprehensive evaluation of the relative efficacy of multiple exercise interventions, even in the absence of sufficient head-to-head comparative studies between different exercise regimens (Caldwell et al., 2005). By synthesizing existing RCT evidence, this study evaluated the comparative effects of 12 exercise interventions on several outcomes, including 6-minute walk distance (6MWD), pulse wave velocity (PWV), and lipid profiles, specifically total cholesterol (TC), triglycerides (TG), high-density lipoprotein cholesterol (HDL-C), and low-density lipoprotein cholesterol (LDL-C), in patients with ASCVD. This study seeks to establish a comprehensive comparative network across various interventions, assess the comparative efficacy of diverse exercise prescriptions, and provide comparative evidence to inform more targeted exercise prescriptions, thereby optimizing long-term management strategies for patients with ASCVD.

## 2 Materials and methods

### 2.1 Search strategy

The study rigorously adhered to standard search procedures for systematic reviews and meta-analyses, with the search conducted up to February 13, 2026. A systematic search was executed using a combination of subject headings and free-text terms in the PubMed, Embase, Cochrane Library, and Web of Science databases to identify RCTs concerning exercise therapy for ASCVD. The detailed search strategy is provided in **Supplementary Methods 1**. Literature screening and data extraction were independently performed by two reviewers (CX and SY), with a third reviewer (YS) consulted in cases of discrepancies to ensure consistency in article selection criteria. Initially, the titles and abstracts of the identified articles were screened to exclude irrelevant studies. Subsequently, the full texts of the remaining articles were evaluated to determine their eligibility based on the inclusion criteria. The study selection process followed the Preferred Reporting Items for Systematic Reviews and Meta-Analyses (PRISMA) guidelines. The meta-analysis protocol has been registered with PROSPERO (No.CRD420261412964). The revised literature search was conducted on June 10, 2026. Full search strings for all databases and details of the updated search are provided in the **Supplementary Methods 1**.

### 2.2 Inclusion and exclusion criteria

#### 2.2.1 Inclusion criteria

Patients diagnosed with ASCVD-related conditions such as coronary heart disease, peripheral arterial disease, or carotid atherosclerosis. There are no restrictions on patient ethnicity or geographic location. Diagnostic criteria are based on the 2019 European Society of Cardiology/European Atherosclerosis Society (ESC/EAS) Guidelines for the Management of Dyslipidemias: Lipid Modification to Reduce Cardiovascular Risk (Mach et al., 2020) (Doi et al., 2023); Intervention: The experimental group must implement at least one exercise intervention while maintaining standard ASCVD treatment measures; the control group receives standard ASCVD treatment measures, including health education and general care (Toval et al., 2025); Outcome measures include PWV, 6MWD, TC, TG, HDL-C, and LDL-C (Makover et al., 2022); All included studies must be RCTs (Bäck et al., 2019); All participants must be 18 years of age or older.

#### 2.2.2 Exclusion criteria

(Grundy et al., 2019) Studies containing duplicate articles or information; (2) Studies involving animal experiments (Toval et al., 2025); Conference reports, letters, or case reports, as well as articles for which the full text is unavailable (Makover et al., 2022); Articles in which data are presented directly in graphical form, or other articles from which data cannot be obtained.

See **Supplementary Table 1** for specific inclusion and exclusion criteria.

### 2.3 Data extraction and quality assessment

#### 2.3.1 Data extraction

Data extraction and quality assessment were conducted independently by two reviewers (CX and SY), and any discrepancies were resolved by a third reviewer (YS). Data were extracted and subsequently cross-checked. In cases of discrepancy, a third researcher was consulted for a final decision. The extracted data primarily included: Basic information: first author, year of publication, sample size, patient age, and diagnosis. Intervention parameters: specific type of exercise intervention (e.g., AE, RT, BFRT), frequency, intensity, duration of a single exercise session, and total intervention duration. Outcome measures: Baseline and post-intervention data for the 6MWD, PWV, and four lipid parameters (TC, TG, HDL-C, LDL-C) were extracted. The mean and standard deviation of changes relative to baseline were extracted as the primary data sources for this study’s analysis. Specific calculation formulas are detailed in **Supplementary Methods 2**, and the basic definitions of each indicator are provided in **Supplementary Table 2**.

#### 2.3.2 Quality assessment

The Cochrane Risk of Bias Assessment Tool 2.0 was used to assess the quality of the included randomized controlled trials. The assessment covered the following domains: randomization, deviation from the assigned intervention, missing outcome data, measurement of outcomes, and selective reporting. Each study was rated as “low risk,” “concerns,” or “high risk” based on the level of risk.

### 2.4 Statistical analysis

In this study, the mvmeta package in Stata 18.0 was used to perform network and conventional meta-analyses. For continuous outcomes, mean differences (MD) with 95% confidence intervals (CI) were used as effect measures. The Cochrane Risk of Bias Assessment Tool 2.0 was used to assess study quality and risk of bias. Prediction interval plots were used to assess heterogeneity, and local inconsistencies were detected using the node-splitting method. Clinical and methodological characteristics were compared to evaluate the similarity of the evidence network, and outcomes were compared between any two interventions listed in the ranking table. The Surface Under the Cumulative Ranking Curve (SUCRA) was used to probabilistically rank the efficacy measures of interventions. SUCRA is used to probabilistically assess the relative efficacy rankings of different interventions based on the current evidence network; it reflects only “relative likelihood” rather than “absolute clinical advantage.” This metric is an exploratory analytical tool, and its results must be interpreted in conjunction with the statistical significance of pairwise comparisons, study quality, sample size, publication bias, and other factors. It should not be used alone as the definitive basis for selecting the clinically optimal intervention.

Given potential differences among studies in exercise intensity and participant characteristics, this study employed a random-effects model for meta-analysis. For continuous variables, standardized means and their 95% confidence intervals were used as effect measures. Heterogeneity was assessed using the I² statistic: when I² > 50%, significant heterogeneity was considered present, and the sources of heterogeneity were investigated through subgroup analysis or sensitivity analysis (exclusion method).

## 3 Results

### 3.1 Study selection

**Figure 1:**
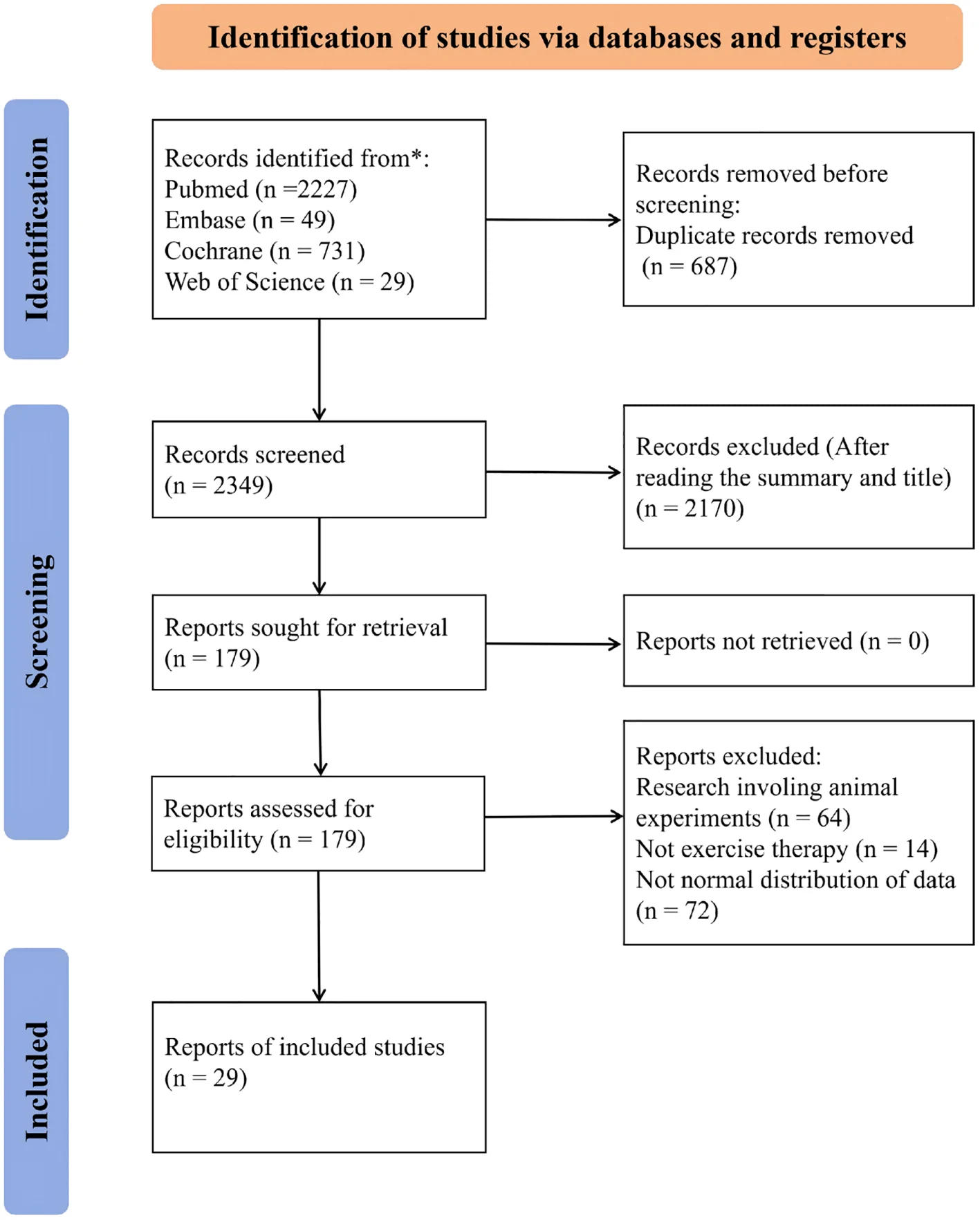
Document screening process and results.

The literature search identified a total of 3,036 potentially relevant articles from the Web of Science, PubMed, Embase, and the Cochrane Library databases. After deduplication and other processes, the collection was narrowed down to 2,349 articles, all of which were in English. Following the predetermined inclusion and exclusion criteria, the initial screening phase involved a detailed review of titles, abstracts, and keywords, ultimately selecting 179 articles for further evaluation. Subsequently, following a comprehensive analysis of the full texts, 29 studies were ultimately selected (Woo et al., 2004; Martins et al., 2010; Bronas et al., 2011; Ohta et al., 2012; Parmenter et al., 2013; Kearney et al., 2014; Oliveira et al., 2015; Mogharnasi et al., 2017; Wong et al., 2018; McDermott et al., 2019; Park et al., 2019; Ratajczak et al., 2019; Correia et al., 2020; Laslovich et al., 2020; Park et al., 2020; Lin et al., 2021; McDermott et al., 2021; Bearne et al., 2022; Baynard et al., 2023; Gardner et al., 2023; Parkington et al., 2023; Ma et al., 2024; Manfredini et al., 2024; Tang et al., 2024; Wang et al., 2024; Zeng et al., 2024; Jia et al., 2025; McDermott et al., 2025; Son et al., 2025). All 29 articles were included in English. The literature screening flowchart is shown in **Figure 1**. The basic characteristics of the included studies are detailed in **Supplementary Table 3**, while specific descriptions of the exercise interventions across all studies are listed in **Supplementary Table 4**.

These studies evaluated 12 types of exercise (including various combinations), covering Conventional Treatment (CT), Aerobic Exercise (AE), Resistance Training (RT), Isometric Handgrip Exercise (IHE), Aerobic Exercise + Resistance Training (AERT), Qigong + Dancing (QD), Supervision + Aerobic Exercise (SAE), Supervision + Resistance Exercise (SRE), Tai Chi Chuan (TCC), Resistance Exercise + Blood Flow Restriction (BFRT), Heat Treat Aerobic Exercise (HTAE), and Stretching Exercise (SE). For detailed definitions and classifications of these exercise therapies, see **Supplementary Table 5**.

### 3.2 Assessment of study quality

This study strictly followed the Cochrane Risk of Bias Assessment Tool 2.0 recommended in the Cochrane Handbook of Systematic Reviews of Interventions to conduct a systematic assessment of risk of bias for the 29 included RCTs (Higgins et al., 2024). The assessment covered five domains: the randomization process, deviation from the intended intervention, missing outcome data, measurement of outcomes, and selection bias in reporting results.

#### 3.2.1 Analysis of bias risk dimensions

The evaluation results for each dimension are as follows:

##### 3.2.1.1 Randomized trials

Regarding randomized trials, a total of 9 studies were rated as low risk (Parmenter et al., 2013; Kearney et al., 2014; Oliveira et al., 2015; Correia et al., 2020; McDermott et al., 2021; Bearne et al., 2022; Gardner et al., 2023; Ma et al., 2024; Manfredini et al., 2024), 20 were rated as having moderate risk (Woo et al., 2004; Martins et al., 2010; Bronas et al., 2011; Ohta et al., 2012; Mogharnasi et al., 2017; Wong et al., 2018; McDermott et al., 2019; Park et al., 2019; Ratajczak et al., 2019; Laslovich et al., 2020; Park et al., 2020; Lin et al., 2021; Baynard et al., 2023; Parkington et al., 2023; Tang et al., 2024; Wang et al., 2024; Zeng et al., 2024; Jia et al., 2025; McDermott et al., 2025; Son et al., 2025). Overall, most studies provide insufficient reporting regarding the generation of random sequences or the allocation of hidden variables; therefore, this dimension is considered to carry a certain level of risk.

##### 3.2.1.2 Outcome data completeness

Regarding outcome data completeness, a total of 26 studies were rated as low risk (Woo et al., 2004; Martins et al., 2010; Bronas et al., 2011; Ohta et al., 2012; Parmenter et al., 2013; Kearney et al., 2014; Oliveira et al., 2015; Mogharnasi et al., 2017; Wong et al., 2018; McDermott et al., 2019; Park et al., 2019; Ratajczak et al., 2019; Correia et al., 2020; Laslovich et al., 2020; Park et al., 2020; Lin et al., 2021; McDermott et al., 2021; Gardner et al., 2023; Parkington et al., 2023; Manfredini et al., 2024; Tang et al., 2024; Wang et al., 2024; Zeng et al., 2024; Jia et al., 2025; McDermott et al., 2025; Son et al., 2025), 3 studies were rated as having some risk (Bearne et al., 2022; Baynard et al., 2023; Ma et al., 2024), no high-risk studies were identified. Overall, outcome data were relatively complete in most studies, but some studies may still have issues with loss to follow-up, withdrawal, or inadequate reporting of outcome data.

##### 3.2.1.3 Outcome measures

Regarding outcome measures, a total of 28 studies were rated as low risk (Woo et al., 2004; Martins et al., 2010; Bronas et al., 2011; Ohta et al., 2012; Parmenter et al., 2013; Kearney et al., 2014; Oliveira et al., 2015; Mogharnasi et al., 2017; Wong et al., 2018; McDermott et al., 2019; Park et al., 2019; Ratajczak et al., 2019; Correia et al., 2020; Park et al., 2020; Lin et al., 2021; McDermott et al., 2021; Bearne et al., 2022; Baynard et al., 2023; Gardner et al., 2023; Parkington et al., 2023; Ma et al., 2024; Manfredini et al., 2024; Tang et al., 2024; Wang et al., 2024; Zeng et al., 2024; Jia et al., 2025; McDermott et al., 2025; Son et al., 2025), 1 study was assessed as having moderate risk (Laslovich et al., 2020), no high-risk studies were identified. Since the 6MWD, PWV, and lipid profile measures included in this study are predominantly objective measurements, the overall risk in this dimension is relatively low.

##### 3.2.1.4 Deviations from the intended intervention

Regarding deviations from the intended intervention, a total of 21 studies were rated as low risk (Woo et al., 2004; Martins et al., 2010; Ohta et al., 2012; Parmenter et al., 2013; Kearney et al., 2014; Oliveira et al., 2015; Mogharnasi et al., 2017; Wong et al., 2018; McDermott et al., 2019; Correia et al., 2020; Laslovich et al., 2020; Lin et al., 2021; McDermott et al., 2021; Bearne et al., 2022; Parkington et al., 2023; Manfredini et al., 2024; Tang et al., 2024; Wang et al., 2024; Zeng et al., 2024; McDermott et al., 2025; Son et al., 2025), 4 studies were rated as having moderate risk (Bronas et al., 2011; Baynard et al., 2023; Gardner et al., 2023; Jia et al., 2025), 4 studies were rated as high risk (Park et al., 2019; Ratajczak et al., 2019; Park et al., 2020; Ma et al., 2024). These results suggest that some studies had shortcomings in the implementation of exercise interventions, adherence monitoring, or the application of blinding. Since it is inherently difficult to achieve complete blinding of both participants and intervention providers in exercise intervention studies, this dimension is a significant source of overall risk of bias.

##### 3.2.1.5 Selection bias in reporting results

Regarding selection bias in reporting results, a total of 16 studies were assessed as having a low risk (Parmenter et al., 2013; Oliveira et al., 2015; Wong et al., 2018; McDermott et al., 2019; Park et al., 2019; Correia et al., 2020; Park et al., 2020; Lin et al., 2021; McDermott et al., 2021; Bearne et al., 2022; Baynard et al., 2023; Parkington et al., 2023; Ma et al., 2024; Manfredini et al., 2024; Tang et al., 2024; McDermott et al., 2025), 13 studies were assessed as having moderate risk (Woo et al., 2004; Martins et al., 2010; Bronas et al., 2011; Ohta et al., 2012; Kearney et al., 2014; Mogharnasi et al., 2017; Ratajczak et al., 2019; Laslovich et al., 2020; Gardner et al., 2023; Wang et al., 2024; Zeng et al., 2024; Jia et al., 2025; Son et al., 2025), no studies were identified as having a high risk. Some studies failed to provide complete trial registration information or pre-published study protocols; therefore, selective reporting bias cannot be completely ruled out.

The overall risk of bias results are as follows:

Based on an assessment across five dimensions, a total of 5 studies were rated as having a low risk of bias (Parmenter et al., 2013; Oliveira et al., 2015; Correia et al., 2020; McDermott et al., 2021; Manfredini et al., 2024), 20 studies were assessed as having moderate risk (Woo et al., 2004; Martins et al., 2010; Bronas et al., 2011; Ohta et al., 2012; Kearney et al., 2014; Mogharnasi et al., 2017; Wong et al., 2018; McDermott et al., 2019; Laslovich et al., 2020; Lin et al., 2021; Bearne et al., 2022; Baynard et al., 2023; Gardner et al., 2023; Parkington et al., 2023; Tang et al., 2024; Wang et al., 2024; Zeng et al., 2024; Jia et al., 2025; McDermott et al., 2025; Son et al., 2025), four studies were overall rated as high risk (Park et al., 2019; Ratajczak et al., 2019; Park et al., 2020; Ma et al., 2024). Overall, there were variations in the methodological quality of the included studies, with primary sources of bias concentrated in the randomization process, deviation from the intended intervention, and selective reporting. Given the inherent limitations of exercise intervention studies regarding blinding and control of intervention adherence, the interpretation of subsequent results should be based on a comprehensive assessment that incorporates sensitivity analyses, publication bias analyses, and study quality.

**Figure 2:**
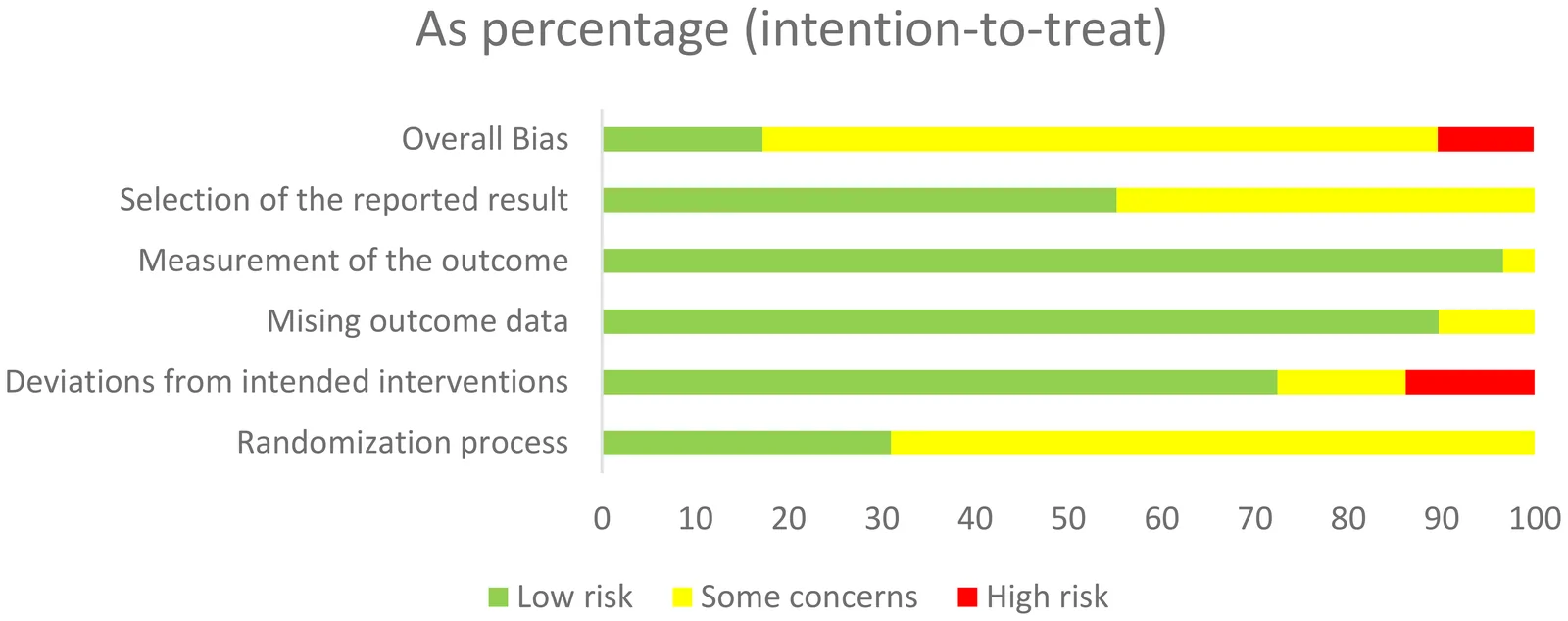
Risk of bias assessment included in the study.

A summary of the risk assessment results across all dimensions and overall is presented in **Figure 2**. The bias risk plots for each study are shown in **Supplementary Figure 1**.

### 3.3 Results of the meta-analysis of primary outcome measures

The 6MWD and PWV are key indicators for assessing exercise capacity and vascular function in patients with ASCVD, reflecting exercise tolerance and arterial stiffness, respectively (Mach et al., 2020). Therefore, this study utilized these measures as functional and vascular-related outcome indicators to comprehensively evaluate the effects of different exercise interventions on improving cardiovascular function in patients.

#### 3.3.1 6MWD

**Figure 3:**
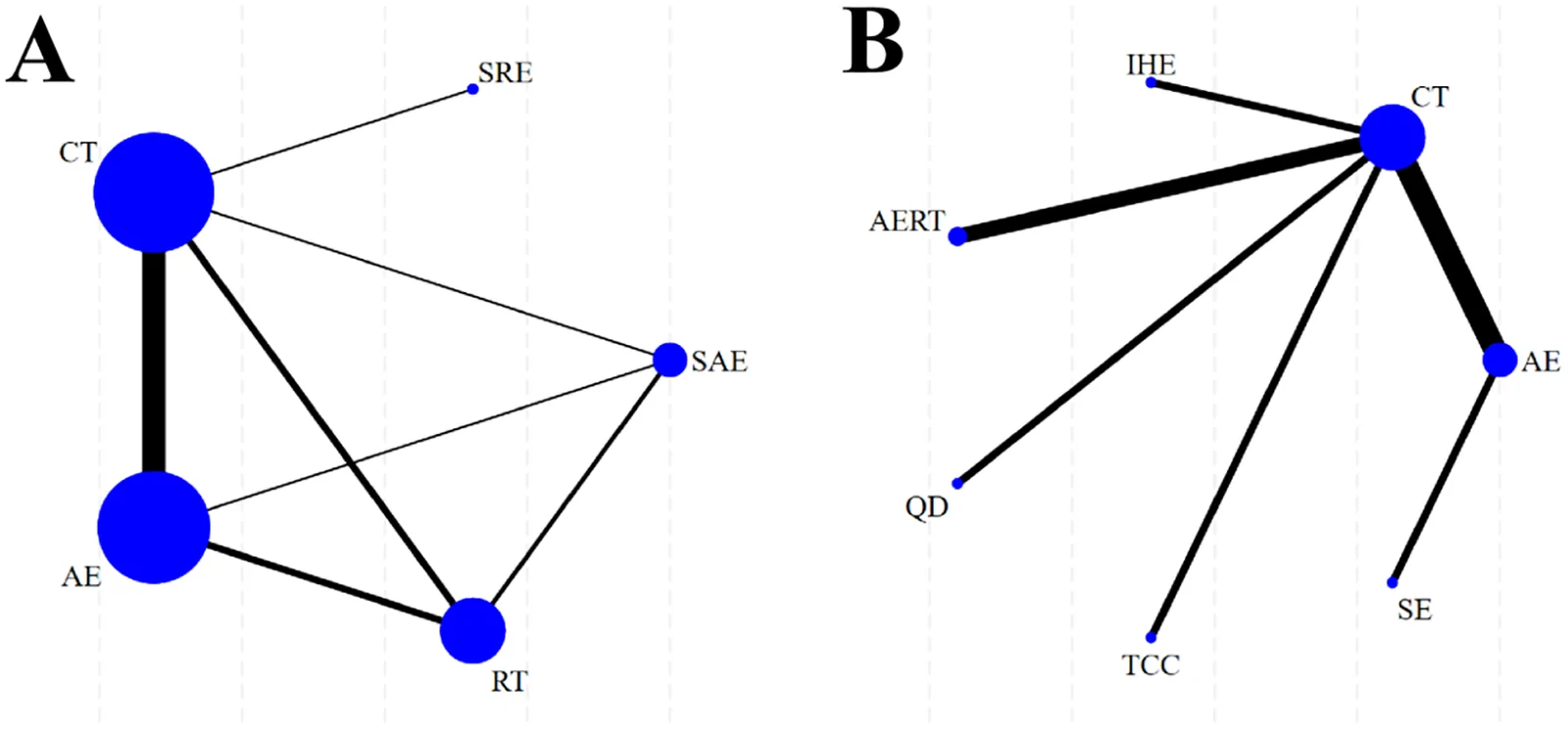
The evidence network of all papers on different treatments. **(A)** 6MWD; **(B)** PWV. The thickness of the lines represents the number of studies, and the sizes of the nodes indicate the total sample sizes for each treatment.

**Figure 4:**
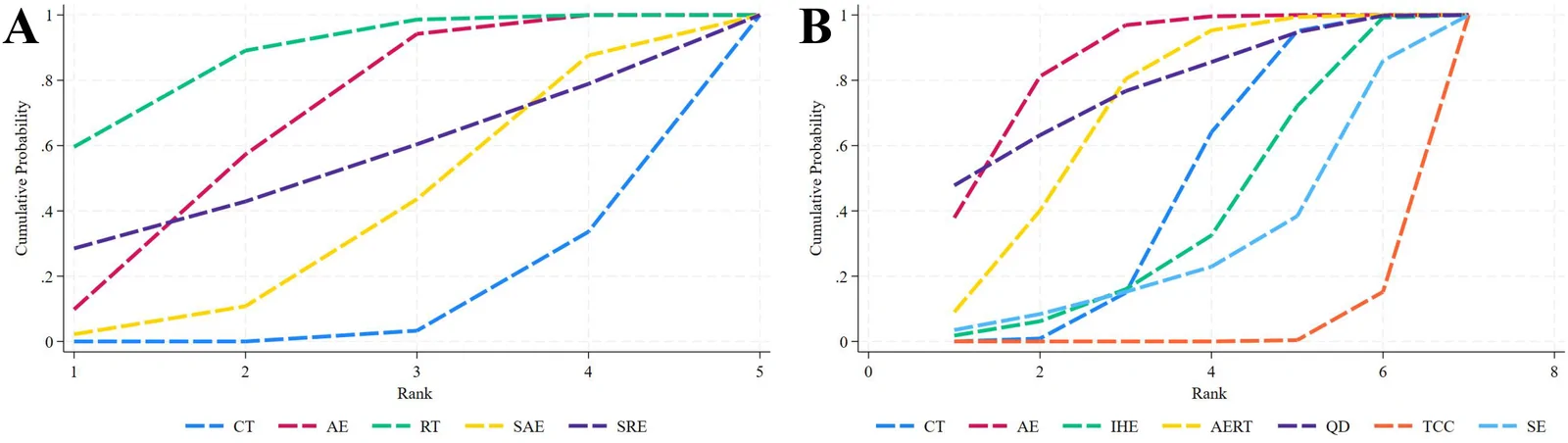
Ranking of the cumulative probabilities for core parameters. **(A)** 6MWD; **(B)** PWV.

A total of 11 studies reported 6MWD outcomes following exercise therapy, involving 1,292 patients and six intervention types (Martins et al., 2010; Bronas et al., 2011; McDermott et al., 2019; Park et al., 2019; Laslovich et al., 2020; Lin et al., 2021; Bearne et al., 2022; Baynard et al., 2023; Gardner et al., 2023; Manfredini et al., 2024; Jia et al., 2025). The network relationships among the interventions are shown in **Figure 3A**. The network contained partial closed loops. As the global inconsistency test yielded P > 0.05, the consistency model was used for the NMA. The NMA showed that two exercise interventions significantly improved 6MWD compared with conventional treatment: AE (MD = 44.30, 95% CI: [16.30, 72.30]) and RT (MD = 56.42, 95% CI: [15.94, 96.91]). The results of pairwise comparisons between groups are presented in **Supplementary Table 6** and **Supplementary Figure 2**. The node-splitting method indicated no significant inconsistencies between nodes, suggesting that the results of direct and indirect comparisons are generally consistent within the network structure of this study. Analysis of the SUCRA ranking results revealed that RT may have the greatest potential to improve patients’ 6MWD (80.6%), followed by AE (65.2%). The SUCRA values and rankings for the remaining therapies are shown in **Supplementary Table 7** and **Figure 4A**.

We proceeded to conduct a comprehensive analysis of the results using conventional meta-analysis methods. The overall pooled results indicated high heterogeneity among studies (I² = 77.70%, *P* < 0.001), therefore, a random-effects model was employed for analysis. Subgroup analysis revealed that AE significantly improved 6MWD (MD = 56.82, 95% CI: [31.01, 82.64], Z = 4.31 *P* < 0.001), with the difference being statistically significant. The pooled forest plot is shown in **Supplementary Figure 3**. Given the high heterogeneity in this study, a sensitivity analysis was conducted by sequentially excluding individual studies (**Supplementary Figure 4**). Overall, after excluding any single study, the direction and statistical significance of the pooled effect size remained largely unchanged, suggesting that the overall conclusions are robust. However, as shown in the forest plot and sensitivity analysis, one specific study exhibited a significant deviation, presenting a relatively high effect size (MD = 113.70) and weight (10.82%) (Lin et al., 2021). After excluding this deviated study, the overall pooled effect size decreased significantly (the MD dropped to 37.26), suggesting that this specific data deviation may have had a certain influence on the overall results and heterogeneity.

#### 3.3.2 PWV

A total of 9 studies reported on patients’ PWV following exercise therapy, involving 564 patients and 6 intervention types (Ohta et al., 2012; Kearney et al., 2014; Oliveira et al., 2015; Wong et al., 2018; Park et al., 2019; Correia et al., 2020; Wang et al., 2024; Zeng et al., 2024; Son et al., 2025). The network relationships among the interventions are shown in **Figure 3B**. Partial closed-loop formation was observed. A consistency model test revealed *P* > 0.05, indicating that a consistency model could be used for the NMA. The NMA showed that, regarding PWV, AE (MD = −1.52, 95% CI [−2.70, −0.34]) demonstrated a significant advantage over CT, with the difference being statistically significant (*P* < 0.05). It should be noted that compared with CT, TCC was associated with elevated PWV levels (MD = 3.79, 95% CI [2.00, 5.59]), and the difference was statistically significant (*P* < 0.05), suggesting that TCC may not be superior in improving PWV. Pairwise comparisons between groups showed that AE was superior to TCC (MD = −5.31, 95% CI [−7.46, −3.17]), isometric handgrip exercise was superior to TCC (MD = −3.19, 95% CI [−5.96, −0.43]), AERT was superior to TCC (MD = −4.65, 95% CI [−6.81, −2.50]), and QD was superior to TCC (MD = −5.32, 95% CI [−9.30, −1.34]), with all differences being statistically significant (*P* < 0.05). The results of pairwise comparisons between groups are presented in the league table and dendrogram in **Supplementary Table 8** and **Supplementary Figure 5**. The node splitting method indicated no significant inconsistencies between nodes, suggesting that the results of direct and indirect comparisons were generally consistent within the network structure of this study. The analysis of the SUCRA ranking results indicated that AE exhibited the highest potential for improving patients’ PWV, with a SUCRA value of 85.9%, followed by QD at 77.9% and AERT at 70.7%. The SUCRA values for the remaining therapies are detailed in **Supplementary Table 9** and **Figure 4B**.

We proceeded to conduct a comprehensive analysis of the results using conventional meta-analysis methods. The overall pooled results indicated high heterogeneity among studies (I² = 95.90%, *P* < 0.001), therefore, a random-effects model was selected for analysis. The results of the pairwise comparison forest plot are shown in **Supplementary Figure 6**. Subgroup analysis demonstrated that AERT significantly reduced PWV levels (MD = −0.87, 95% CI: [−1.26, −0.49], Z = −4.44, *P* < 0.001), indicating a statistically significant difference. In contrast, TCC was associated with an increase in PWV levels (MD = 3.79, 95% CI: [2.98, 4.60], Z = 9.16, *P* < 0.001), suggesting it may not be effective in improving PWV. No statistically significant differences were observed for the other interventions. Given the high heterogeneity in this study, sensitivity analyses were conducted (**Supplementary Figure 7**). Combining the forest plot and sensitivity analysis plots reveals significant deviations in two specific studies. The effect size in one study (MD = −2.28) was significantly lower than that in most other studies, while the direction of effect in another study (MD = 3.79) deviated in the opposite direction (positive) compared to the majority (Wang et al., 2024). In particular, the sensitivity analysis demonstrates that excluding the latter deviated study resulted in a significant shift in the overall pooled effect size, suggesting that this data deviation had a substantial impact on the overall results and heterogeneity; however, since the sensitivity analysis did not provide I² values for each individual exclusion, it is not yet possible to definitively determine that this specific study is the sole source of heterogeneity.

### 3.4 Lipid-related parameters

TC, TG, HDL-C, and LDL-C serve as crucial biomarkers for evaluating lipid metabolism and assessing the risk of atherosclerosis. These indicators reflect alterations in cholesterol levels, triglyceride metabolism, and the presence of anti-atherosclerotic and atherogenic lipoproteins, respectively (Mach et al., 2020). Consequently, this study utilizes these lipid-related outcome measures to assess the impact of various exercise interventions on lipid metabolism and cardiovascular risk factors in patients with ASCVD.

#### 3.4.1 TC

**Figure 5:**
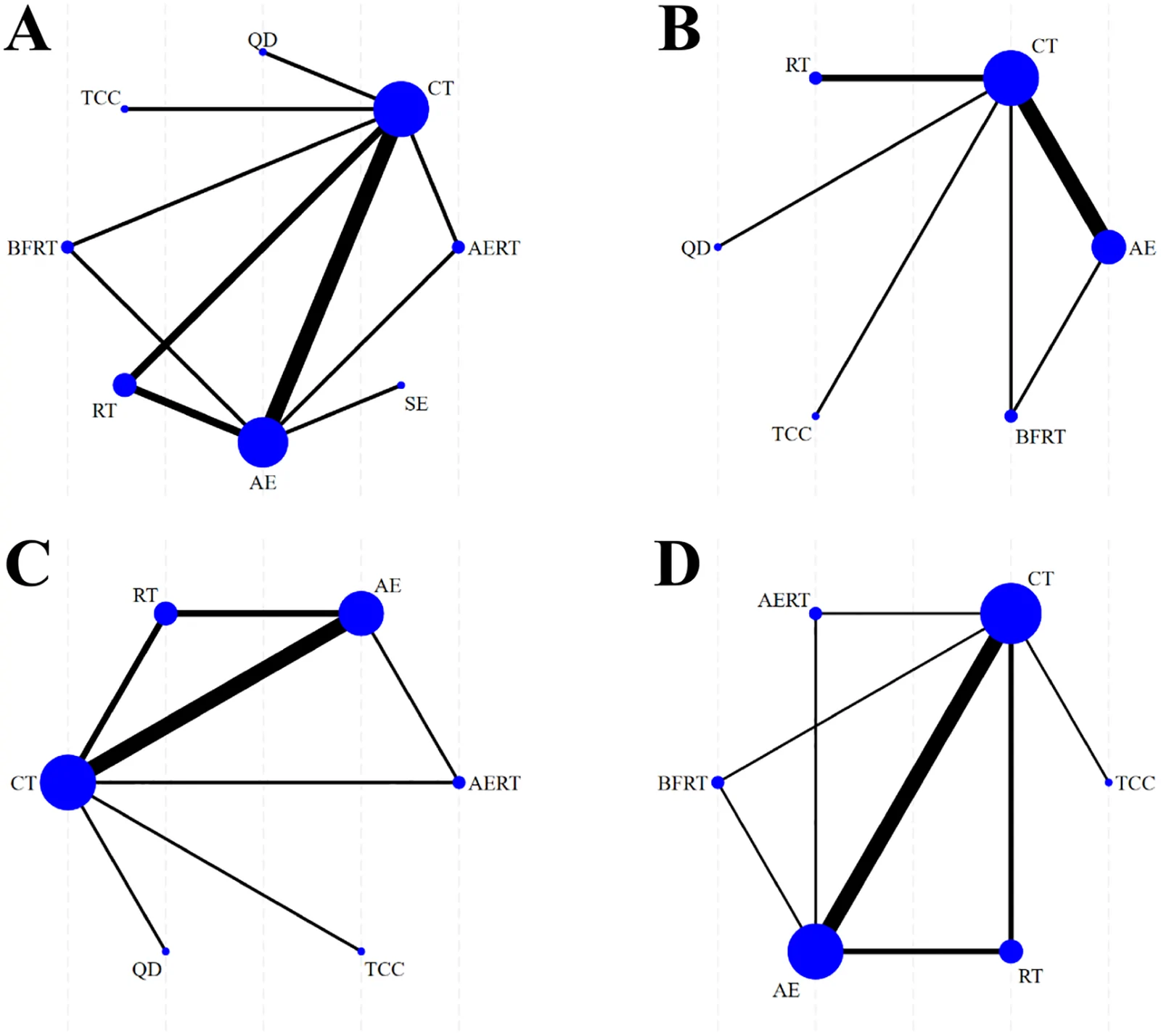
The evidence network of all papers on different treatments. **(A)** TC; **(B)** TG; **(C)** HDL-C; **(D)** LDL-C; the thickness of the lines represents the number of studies, and the sizes of the nodes indicate the total sample sizes for each treatment.

**Figure 6:**
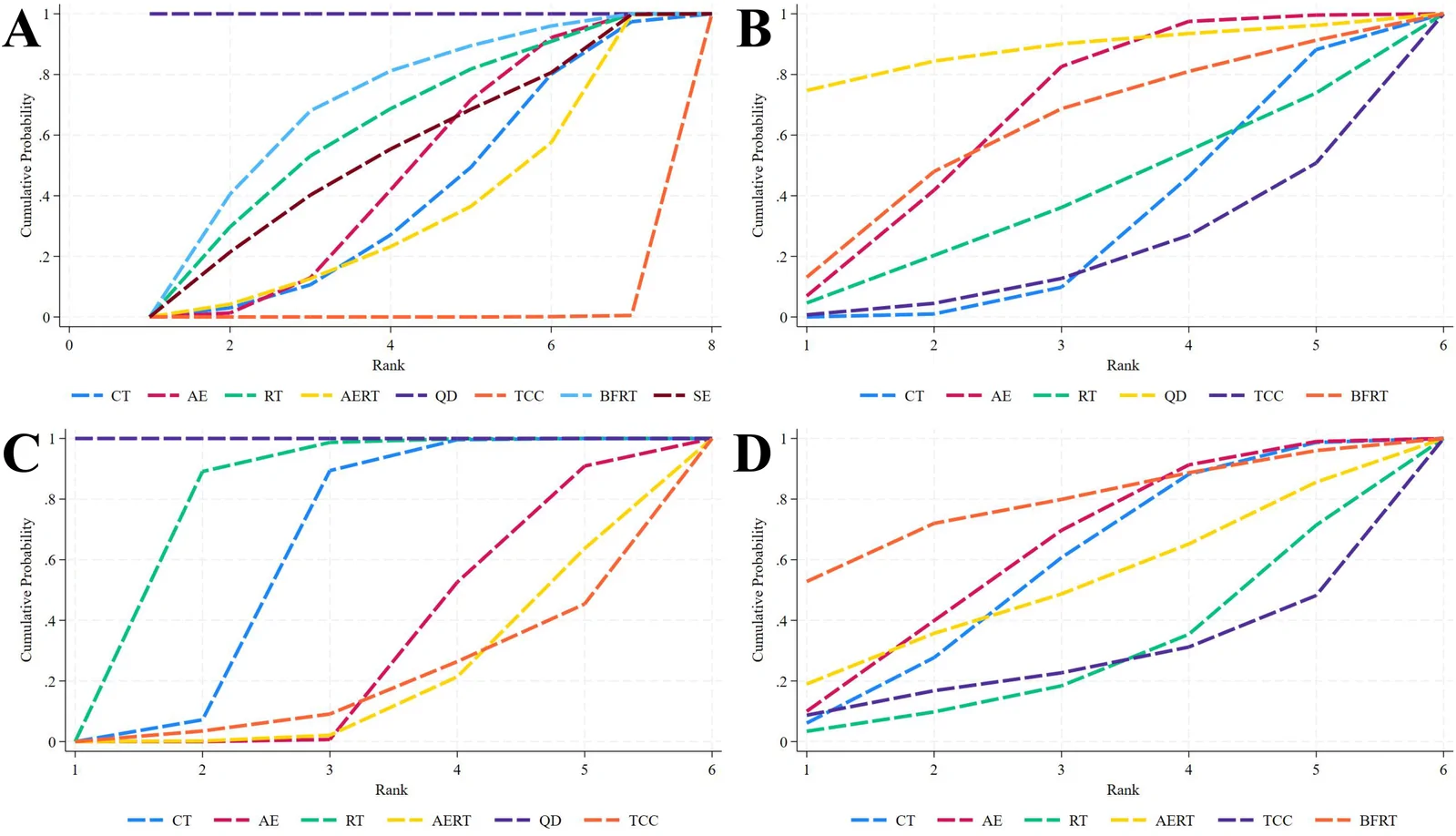
Ranking of the cumulative probabilities for core parameters. **(A)** TC; **(B)** TG; **(C)** HDL-C; **(D)** LDL-C.

A total of 9 studies, encompassing 616 patients and seven different intervention types, reported on TC outcomes following exercise therapy (Woo et al., 2004; Martins et al., 2010; Ohta et al., 2012; Kearney et al., 2014; Mogharnasi et al., 2017; Ratajczak et al., 2019; Wang et al., 2024; Zeng et al., 2024; Li et al., 2022). The network relationships among these interventions are depicted in **Figure 5A**, where partial closed-loop formation was observed. Testing with the inconsistency model yielded a P-value greater than 0.05, suggesting that a consistent model could be employed for the NMA. The NMA indicated that, in terms of TCC, the intervention QD (mean difference [MD] = −1.46, 95% CI [−1.99, −0.93]) demonstrated a significant advantage over CT, with the difference being statistically significant (*P* < 0.05). It should be noted that the TCC intervention actually increased TC levels compared to CT (MD = 1.12, 95% CI [0.43, 1.81]), and the difference was statistically significant, suggesting that it may not be superior in reducing TC. Pairwise comparisons between groups showed that QD was superior to AE (MD = −1.41, 95% CI [−2.04, −0.79]), QD was superior to RT (MD = −1.31, 95% CI [−2.00, −0.62]), QD was superior to AERT (MD = −1.53, 95% CI [−2.16, −0.89]), BFRT was superior to TCC (MD = −1.34, 95% CI [−2.17, −0.50]), stretching exercise was superior to TCC (MD = −1.22, 95% CI [−2.10, −0.33]), and these differences were statistically significant (*P* < 0.05). The results of pairwise comparisons between groups are presented in the league table and dendrogram in **Supplementary Table 10** and **Supplementary Figure 8**. Node splitting analysis indicated no significant inconsistencies between nodes, suggesting that the results of direct and indirect comparisons were generally consistent within the study’s network structure. Analysis of the SUCRA ranking revealed that QD may have the greatest potential to reduce patients’ TC levels (100%), followed by BFRT (67.9%). SUCRA values for the remaining therapies are shown in **Supplementary Table 11** and **Figure 6A**.

We proceeded to conduct a comprehensive analysis of the results using conventional meta-analysis methods. The overall pooled results revealed substantial heterogeneity among studies (I² = 76.00%, *P* < 0.001), warranting the use of a random-effects model for analysis. The results of the pairwise comparison forest plot are shown in **Supplementary Figure 9**. Further subgroup analysis indicated that QD significantly reduced TC levels (MD = −1.46, 95% CI: [−1.99, −0.93], Z = −5.37, *P* < 0.001), whereas the TCC intervention resulted in elevated TC levels (MD = 1.12, 95% CI: [0.43, 1.81], Z = 3.20, *P* = 0.001), suggesting it may not be effective for lowering TC. Differences in the remaining exercise interventions were not statistically significant. Notably, the results for QD exercise were consistent with those of the NMA, indicating a certain degree of robustness in our study. Given the high heterogeneity in this study, we conducted further sensitivity analyses (**Supplementary Figure 10**). Combining the forest plot and sensitivity analysis plots reveals significant deviations in two specific studies. The effect size in one study (MD = −1.46) was significantly lower than that in other studies (Zeng et al., 2024), while the direction of effect in another study (MD = 1.12) deviated in the opposite direction (positive) compared to most studies (Wang et al., 2024). In particular, the sensitivity analysis demonstrates that excluding either of these two deviated studies resulted in a significant shift in the overall pooled effect size, suggesting that these data deviations had a substantial impact on the overall results and heterogeneity. However, since the sensitivity analysis did not provide I² values for each individual exclusion, it is not yet possible to definitively determine that they are the sole source of heterogeneity.

#### 3.4.2 TG

A total of 6 studies reported on TG levels in patients following exercise therapy, involving 479 patients and five types of interventions (Ohta et al., 2012; Kearney et al., 2014; Mogharnasi et al., 2017; Tang et al., 2024; Zeng et al., 2024; Li et al., 2022). The network relationships among the interventions are shown in **Figure 5B**. Partial closed loops were formed, testing using an inconsistency model yielded *P* > 0.05, so a consistency model was used for the NMA. The NMA showed that, regarding TG, compared with CT, the various exercise interventions were associated with the following effects: AE (MD = −0.25, 95% CI [−0.51, 0.00]), RT (MD = −0.04, 95% CI [−0.67, 0.58]), QD (MD = −0.84, 95% CI [−2.00, 0.32]), TCC (MD = 0.12, 95% CI [−0.34, 0.58]), and BFRT (MD = −0.26, 95% CI [−0.88, 0.36]), with none of these differences being statistically significant (*P* > 0.05). Among these, AE and BFRT exhibited a trend toward lowering TG levels, however, their 95% CI either encompassed or were close to 0, precluding any definitive statistical advantage at this time. The results of pairwise comparisons between groups are presented in **Supplementary Table 12** and **Supplementary Figure 11**. Node splitting analysis indicated no significant inconsistencies between nodes, suggesting that the results of direct and indirect comparisons were generally consistent within the network structure of this study. Analysis of the SUCRA ranking results revealed that QD may have the greatest potential to reduce patients’ TG levels (87.6%), followed by AE (65.3%). SUCRA values for the remaining therapies are shown in **Supplementary Table 13** and **Figure 6B**. However, since SUCRA is based solely on indirect comparisons, interpretation of these results should be cautious, and the focus should be on exploring the statistical significance among the various interventions.

We proceeded to conduct a comprehensive analysis of the results using conventional meta-analysis methods. The pooled results demonstrated substantial heterogeneity among the studies (I² = 80.16%, *P* < 0.001), necessitating the use of a random-effects model for analysis. Pairwise comparisons indicated that exercise interventions did not significantly reduce TG levels (MD = −0.18, 95% CI: [−0.39, 0.03], Z = −1.67, *P* = 0.10), as depicted in the pairwise comparison forest plot in **Supplementary Figure 12**. Subgroup analysis indicated no statistically significant differences in the effects of various exercise interventions on TG levels. Given the high heterogeneity in this study, a sensitivity analysis was further conducted (**Supplementary Figure 13**). Combining the forest plot and sensitivity analysis plot reveals significant deviations in two specific studies. The effect size of one study (MD = −0.56) was significantly lower than that of other studies and accounted for a large proportion of the total weight (18.88%) (Tang et al., 2024), while the direction of the effect in another study (MD = 0.12) deviated in the opposite direction (positive) compared to most studies (Wang et al., 2024). In particular, the sensitivity analysis specifically illustrates that the exclusion of the previously identified deviant study led to a notable alteration in the overall pooled effect size. This suggests that the deviation in data may have significantly influenced the overall results and contributed to heterogeneity (Tang et al., 2024). Nevertheless, the absence of I² values for each individual exclusion in the sensitivity analysis precludes a definitive conclusion that this particular study is the sole contributor to heterogeneity.

#### 3.4.3 HDL-C

A total of 9 studies reported on HDL-C levels in patients following exercise therapy, involving 690 patients and five types of interventions (Woo et al., 2004; Martins et al., 2010; Ohta et al., 2012; Kearney et al., 2014; Mogharnasi et al., 2017; Ratajczak et al., 2019; Tang et al., 2024; Wang et al., 2024; Zeng et al., 2024). The network relationships among the interventions are shown in **Figure 5C**. Partial closed-loop formation was observed. Since the inconsistency model test yielded *P* > 0.05, a consistency model was used for the NMA. The NMA revealed that, regarding HDL-C, one exercise intervention demonstrated a significant advantage over CT, specifically QD (MD = 0.61, 95% CI [0.38, 0.84]), with the difference being statistically significant (*P* < 0.05). It should be noted that AE (MD = −0.12, 95% CI [−0.22, −0.03]) actually reduced HDL-C levels compared to CT, and the difference was statistically significant (*P* < 0.05). Pairwise comparisons between groups showed that RT was superior to AE (MD = 0.22, 95% CI [0.08, 0.37]), QD was superior to AE (MD = 0.73, 95% CI [0.49, 0.98]), QD was superior to RT (MD = 0.51, 95% CI [0.24, 0.77]), QD was superior to AERT (MD = 0.79, 95% CI [0.50, 1.07]), and all differences were statistically significant (*P* < 0.05). The results of pairwise comparisons between groups are presented in the league table and dendrogram in **Supplementary Table 14** and **Supplementary Figure 14**. Node splitting analysis indicated no significant inconsistencies between nodes, suggesting that the results of direct and indirect comparisons were generally consistent within the network structure of this study. Analysis of the SUCRA ranking revealed that QD may have the greatest potential to increase patients’ HDL-C levels (100%), followed by RT (77.5%). SUCRA values for the remaining therapies are shown in **Supplementary Table 15** and **Figure 6C**.

We proceeded to conduct a comprehensive analysis of the results using conventional meta-analysis methods. The overall pooled results indicated high heterogeneity among studies (I² = 90.88%, *P* < 0.001), therefore, a random-effects model was selected for analysis. Pairwise comparisons indicated that the overall impact of exercise on HDL-C levels was not statistically significant (MD = 0.00, 95% CI: [−0.16, 0.17], Z = 0.03, *P* = 0.98). The corresponding pairwise comparison forest plot is presented in **Supplementary Figure 15**. Subgroup analysis demonstrated that QD (MD = 0.61, 95% CI: [0.44, 0.78], Z = 6.85, *P* < 0.001) significantly elevated HDL-C levels, whereas AERT (MD = −0.20, 95% CI: [−0.34, −0.06], Z = −2.73, *P* = 0.01) was linked to a statistically significant reduction in HDL-C levels. No statistically significant differences were observed for the other exercise interventions. The results for QD exercise were consistent with those of the NMA, suggesting that our study possesses a certain degree of robustness. Given the high heterogeneity in this study, we conducted further sensitivity analyses (**Supplementary Figure 16**). Combining the forest plot and sensitivity analysis plots reveals significant deviations in two specific studies. The effect size in one study (MD = −0.22) was markedly negative (Tang et al., 2024), whereas the effect direction in another study (MD = 0.61) deviated significantly in the positive direction with a substantial magnitude (Zeng et al., 2024). The sensitivity analysis notably indicates that the exclusion of either of the two outlier studies results in a significant alteration of the overall pooled effect size. This suggests that these data deviations may have substantially influenced the overall results and contributed to heterogeneity. However, due to the absence of I² values for each individual exclusion in the sensitivity analysis, it remains inconclusive whether these deviations are the sole contributors to heterogeneity.

#### 3.4.4 LDL-C

A total of 9 studies reported on patients’ LDL-C levels following exercise therapy, involving 650 patients across five intervention types (Woo et al., 2004; Martins et al., 2010; Ohta et al., 2012; Kearney et al., 2014; Mogharnasi et al., 2017; Ratajczak et al., 2019; Tang et al., 2024; Wang et al., 2024; Li et al., 2022). The network relationships among the interventions are shown in **Figure 5D**. Since some closed loops were formed and the inconsistency model test yielded *P* > 0.05, a consistency model was used to conduct the NMA. The NMA revealed that, regarding LDL-C, there were no statistically significant differences among the various exercise interventions compared to control (*P* > 0.05). Among them, AE and BFRT showed a trend toward lowering LDL-C levels, however, their 95% CIs both crossed the zero line, precluding any definitive statistical advantage. The results of pairwise comparisons between groups are presented in **Supplementary Table 16** and **Supplementary Figure 17**. The node splitting method indicated no significant inconsistencies among nodes, suggesting that the results of direct and indirect comparisons were generally consistent within the study’s network structure. Analysis of the SUCRA ranking revealed that BFRT may have the greatest potential to reduce patients’ LDL-C levels (77.6%), followed by AE (62.0%). SUCRA values for the remaining therapies are detailed in **Supplementary Table 17** and **Figure 6D**.

We proceeded to conduct a comprehensive analysis of the results using conventional meta-analysis methods. The overall pooled results indicated high heterogeneity among studies (I² = 94.99%, *P* < 0.001), therefore, a random-effects model was selected for analysis. Pairwise comparisons revealed that the overall effect of exercise intervention on LDL-C levels was not statistically significant (MD = 0.05, 95% CI: [−0.36, 0.47], Z = 0.24, *P* = 0.81), with the results depicted in the pairwise comparison forest plot in **Supplementary Figure 18**. A subgroup analysis indicated that BFRT significantly reduced LDL-C levels (MD = −0.49, 95% CI: [−0.80, −0.18], Z = −3.09, *P* = 0.002), whereas TCC was associated with an increase in LDL-C levels (MD = 0.47, 95% CI: [0.17, 0.77], Z = 3.07, *P* = 0.002), suggesting that TCC may not be superior in lowering LDL-C. Differences in the other exercise interventions were not statistically significant. The findings for BFRT were consistent with those from the NMA, suggesting a degree of robustness in our study. Given the high heterogeneity observed, a sensitivity analysis was performed (**Supplementary Figure 19**). The combination of the forest plot and sensitivity analysis plot highlights a significant deviation in one specific study (Tang et al., 2024), where the effect size (MD = −1.40) was notably negative and large. This contrasts sharply with other studies, which exhibited clearly positive effects (e.g., MD = 0.47) (Wang et al., 2024). Notably, the sensitivity analysis revealed that excluding the previously identified outlier study resulted in the most substantial change in the overall pooled effect size, indicating that this data deviation significantly influenced the overall results and contributed to heterogeneity. However, due to the absence of I² values for each individual exclusion in the sensitivity analysis, it remains inconclusive whether this particular study is the sole source of heterogeneity.

### 3.5 Assessment of publication bias and small sample size effects

**Figure 7:**
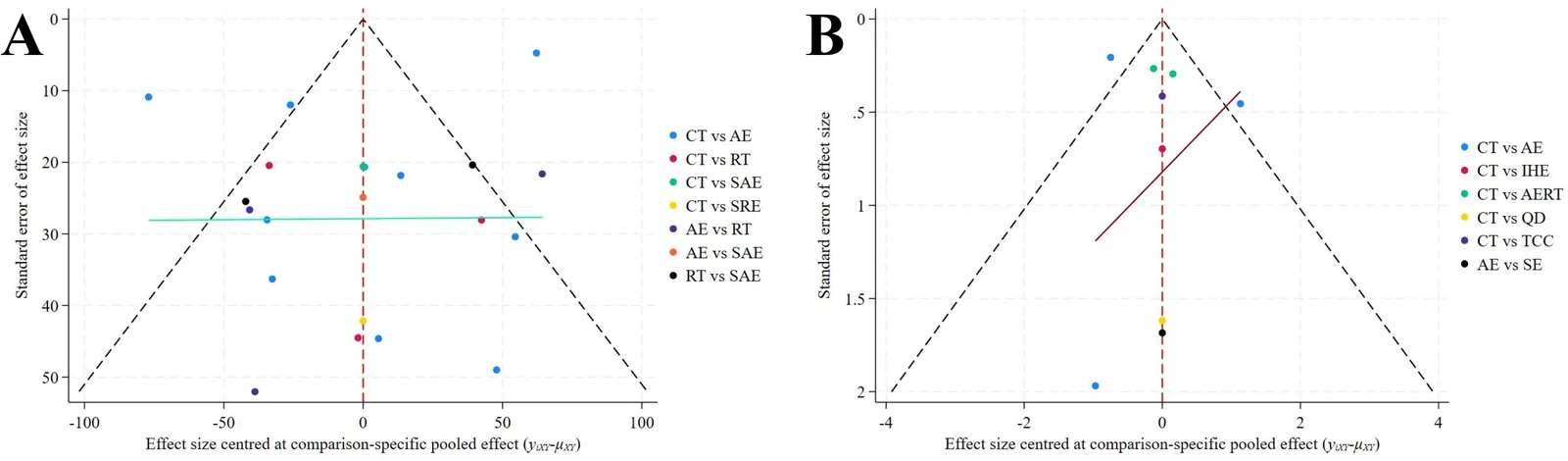
Funnel plot of cardiovascular indicators. **(A)** 6MWD; **(B)** PWV.

**Figure 8:**
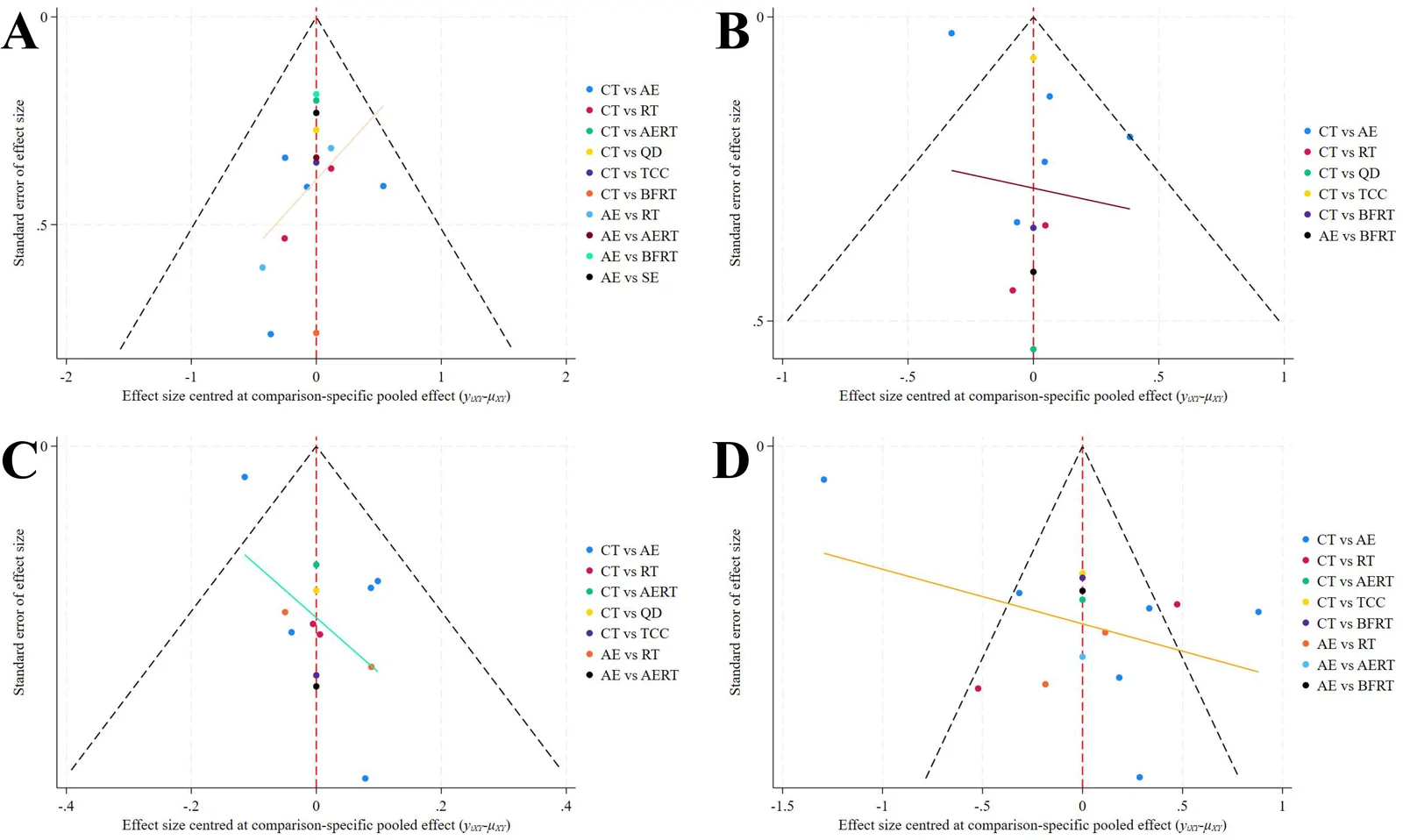
Lipid profile funnel chart. **(A)** TC; **(B)** TG; **(C)** HDL-C; **(D)** LDL-C.

To assess potential small-study effects and publication bias, comparison-adjusted funnel plots were constructed for each outcome, as shown in **Figure 7** and **Figure 8**. For 6MWD, although several estimates were located away from the central line, the fitted regression line was approximately parallel to the X-axis, and no marked directional asymmetry was observed. In contrast, the PWV funnel plot showed an uneven distribution of estimates and a clearly sloping fitted regression line, suggesting possible small-study effects. However, this finding should be interpreted cautiously because of the limited number of studies.

Among the lipid-related outcomes, most TC estimates were located near the central line, although the fitted regression line showed some deviation from the horizontal, indicating possible mild asymmetry. For TG, the estimates were distributed on both sides of the central line, with a modest deviation of the fitted regression line from the horizontal. Most HDL-C estimates were clustered near the central region; however, one estimate deviated toward the negative side, and the fitted regression line was not fully horizontal. The LDL-C funnel plot showed relatively dispersed estimates and a clearly sloping fitted regression line, indicating more apparent asymmetry.

Overall, the comparison-adjusted funnel plots showed varying degrees of asymmetry across the assessed outcomes, particularly for PWV and LDL-C. These patterns suggest that some results may have been affected by small-study effects or publication bias. However, visual asymmetry alone cannot distinguish publication bias from other possible causes, such as heterogeneity, chance, or differences among intervention comparisons.

These findings should be considered when interpreting the results of the NMA and the intervention rankings. The rankings should be evaluated together with the corresponding effect estimates and confidence intervals, as a higher SUCRA value indicates a greater probability of achieving a higher rank but does not necessarily demonstrate statistically significant or clinically meaningful superiority over other interventions. Interpretation should also take into account the number and sample sizes of the included studies, the availability of direct comparative evidence, and the methodological quality of the studies.

## 4 Discussion

### 4.1 Overview of key findings

This study systematically evaluated the effects of different exercise interventions on functional, vascular, and lipid-related outcomes in patients with ASCVD. Overall, exercise interventions improved exercise tolerance, peripheral circulation, arterial stiffness, and selected lipid parameters. However, the magnitude and direction of these effects varied across exercise modalities and outcomes.

For functional outcomes, AE and RT significantly improved the 6MWD, suggesting potential benefits for lower-limb function, cardiorespiratory endurance, and daily activity. Some exercise modalities also showed potential benefits for PWV, possibly through improved vasodilation and reduced arterial stiffness. In contrast, the overall effects of exercise on TG, HDL-C, and LDL-C were not statistically significant in the pairwise meta-analyses. This may partly reflect the widespread use of lipid-lowering medications in patients with ASCVD, which could reduce the additional observable effects of exercise. Nevertheless, the subgroup and network meta-analyses suggested that QD and BFRT may provide specific benefits for selected lipid outcomes. These findings indicate that the metabolic effects of exercise may depend on exercise type, intensity, duration, and patients’ baseline characteristics.

### 4.2 Comparison with other studies

Previous meta-analyses examining the relationship between exercise interventions and cardiovascular health have established a crucial theoretical and evidence-based framework for the current study. For instance, one meta-analysis concentrated on patients with peripheral artery disease, systematically summarizing the positive effects of exercise training on functional outcomes such as cardiorespiratory fitness, pain-free walking distance, and total walking distance. This work effectively highlighted the clinical significance of exercise rehabilitation in managing these patients (Parmenter et al., 2015). Building upon this foundational research, the present study investigates the relative effects of various exercise modalities by integrating multiple exercise patterns within a unified evidence network for comparative analysis. It aims to advance the understanding from the general efficacy of exercise interventions to the more nuanced inquiry of selecting specific exercise modalities tailored to distinct treatment objectives. This analytical approach provides additional evidence for refining clinical exercise prescriptions and elucidates potential differences among various exercise types in terms of functional improvement, vascular function, and metabolic regulation.

A previous NMA in patients with coronary artery disease compared the effects of different exercise types and delivery methods on quality of life, mental health, cognition, and sleep (Toval et al., 2025). This study provided an important methodological reference for the comparative evaluation of exercise interventions.

In contrast, the present study focused on functional capacity, vascular function, and lipid metabolism in the broader ASCVD population. The findings were examined using pairwise meta-analysis, sensitivity analysis, network meta-analysis, and comparison-adjusted funnel plots. The main contribution of this study is the provision of comparative evidence for goal-oriented exercise prescriptions. Specifically, different exercise modalities were evaluated according to clinical objectives such as improving exercise tolerance, peripheral perfusion, arterial stiffness, and lipid management.

These findings may inform the design of future head-to-head randomized controlled trials and support the development of more individualized exercise rehabilitation strategies.

### 4.3 Mechanism analysis

The results of this study reveal that the effects of various exercise modalities on different outcome measures in patients with ASCVD are not entirely consistent, indicating a potential “outcome specificity” in exercise interventions. Overall, exercise may exert its beneficial effects through multiple mechanisms, including the enhancement of skeletal muscle function, improvement of vascular endothelial relaxation capacity, regulation of autonomic nervous system balance, reduction of chronic inflammatory responses, and improvement of lipid metabolism.

Specifically, RT may offer distinct advantages in enhancing exercise tolerance. Patients with ASCVD frequently experience limited mobility, reduced lower limb muscle strength, and inadequate muscular endurance. Given that RT primarily utilizes anaerobic glycolysis and the phosphagen system for energy, it can promote the recruitment and hypertrophy of type II muscle fibers, thereby improving skeletal muscle strength and functional activity capacity (Koh et al., 2022). Furthermore, RT has been shown to potentially elevate skeletal muscle glucose transporter type 4 expression, enhance insulin sensitivity, and improve muscle metabolic capacity (Cui et al., 2020). Consequently, improvements in functional indicators such as the 6MWD are likely attributable to enhancements in lower limb muscle strength, endurance, and metabolic function (Blears et al., 2021). However, the impact of RT on vascular stiffness appears to be variable, potentially due to transient elevations in blood pressure and increased mechanical load on the vascular walls during training (Huang et al., 2025).

In contrast, the physiological basis for the improvements in vascular function and metabolic status induced by AE is well-established. AE increases shear stress on blood vessels, stimulates endothelial nitric oxide synthase activity, and enhances nitric oxide bioavailability, thereby improving vasodilation. Concurrently, AE reduces oxidative stress levels and mitigates damage caused by reactive oxygen species to the vascular endothelium. At the metabolic level, AE activates the AMPK-PGC-1α pathway, promotes mitochondrial biogenesis and fatty acid oxidation, and enhances the utilization of glucose and fatty acids in skeletal muscle (Pesta et al., 2017). Consequently, AE is theoretically associated with positive effects on exercise tolerance, PWV, and specific lipid profiles. Nevertheless, the enhancement in lipid profiles observed with AE in this study did not achieve statistical significance. This indicates that for patients with ASCVD who are already under stringent pharmacological management, conventional-intensity AE may be inadequate for eliciting significant lipid remodeling. Moreover, substantial variability in the intensity, frequency, and duration of AE across different studies has resulted in inconsistent findings. Future investigations should consider examining the potential benefits of higher-intensity aerobic exercise, such as high-intensity interval training (HIIT), on lipid metabolism.

Additionally, mind-body exercises like TCC and QD may enhance ASCVD-related indicators through an integrative “body-breath-mind” regulation model. These exercises typically involve low-intensity, slow movements, and specific breathing patterns, which may facilitate vasodilation and reduce arterial stiffness by enhancing vagal tone, decreasing sympathetic nervous system activity, and improving heart rate variability and baroreflex sensitivity (Bower and Irwin, 2016; Tang et al., 2022). Simultaneously, mind-body exercises may alleviate vascular inflammatory damage by decreasing levels of pro-inflammatory factors, including IL-6 and TNF-α (Irwin et al., 2013). These exercises may also impede atherosclerosis-related inflammatory processes by inhibiting the activation of the NOD-like receptor family pyrin domain-containing 3 (NLRP3) inflammasome through the vagal-cholinergic anti-inflammatory pathway (Tang et al., 2022). Additionally, QD and TCC emphasize the synchronization of breathing, movement, and intention, potentially reducing cortisol levels associated with chronic stress by modulating the activity of the hypothalamic-pituitary-adrenal axis. This modulation may subsequently influence the processes of cholesterol synthesis, transport, and degradation.

Moreover, BFRT may exert its effects via a “low-load, high-metabolic stress” mechanism (Vogel et al., 2020). This approach induces transient local hypoxia and the accumulation of metabolic byproducts under low exercise loads, thereby promoting muscle recruitment, vascular adaptation, and localized metabolic responses (Li et al., 2022). The local hypoxia may also activate pathways such as hypoxia-inducible factor-1 alpha/vascular endothelial growth factor (HIF-1α/VEGF), enhancing microcirculation and facilitating the formation of collateral circulation (Blears et al., 2021). Simultaneously, BFRT may promote the recruitment of type I muscle fibers and mitochondrial biogenesis, thereby facilitating specific muscular and metabolic adaptations under conditions of low mechanical load (Preobrazenski et al., 2021). Consequently, BFRT may possess clinical utility for patients with ASCVD who are unable to engage in high-intensity training. However, due to the limited number of studies conducted thus far, further investigation is necessary to elucidate its mechanisms of action and to confirm its clinical efficacy.

Different exercise modalities may influence functional, vascular, and metabolic outcomes through distinct physiological pathways. RT primarily improves muscle strength and physical performance, whereas AE mainly affects cardiorespiratory fitness, endothelial function, and energy metabolism. Mind-body exercises such as TCC and QD may promote vascular homeostasis through autonomic, neuroendocrine, and anti-inflammatory mechanisms. BFRT may produce benefits through local metabolic stress and vascular adaptation.

Therefore, exercise prescriptions for patients with ASCVD should not merely address whether patients should exercise. Instead, targeted exercise modalities should be selected according to specific therapeutic objectives, patients’ exercise tolerance, and safety considerations.

### 4.4 Limitations and future directions

Firstly, there exists heterogeneity in exercise parameters. The studies included exhibited significant variations in exercise intensity, frequency, and duration, ranging from moderate-intensity exercise three times a week to daily TCC practice. Such variations, even within the same type of exercise, may obscure the true effects. Secondly, there are limitations concerning population representativeness. The studies predominantly focused on Asian and European populations, and racial differences in exercise responses, such as β-adrenergic receptor polymorphisms, may constrain the generalizability of the findings. Thirdly, there are limitations related to outcome measures. The studies lacked hard endpoints, such as cardiovascular mortality and myocardial infarction recurrence rates. Instead, surrogate endpoints like the 6MWD and PWV were utilized, and their association with long-term prognosis necessitates further validation.

Recommendations for future research include (1): conducting RCTs to compare various exercise modalities, with a particular focus on direct comparisons between TCC/QD and AE, as well as between BFRT and RT (2); standardizing the reporting of exercise parameters according to the FITT-VP principles: frequency, intensity, time, type, volume, and progression—to minimize protocol heterogeneity (3); incorporating hard endpoints and quality-of-life measures to establish a robust chain of evidence regarding the long-term benefits of exercise interventions (4); investigating the interactive effects of combined “exercise-medication” prescriptions to develop optimal strategies for the comprehensive management of ASCVD.

## 5 Conclusion

Drawing on evidence from randomized controlled trials, this study compares multiple exercise modalities within a unified analytical framework. Its clinical value lies not only in estimating the relative effects of different interventions but also in supporting a more individualized approach to exercise management in patients with ASCVD. Rather than simply advising patients to increase their physical activity, exercise prescriptions should take into account therapeutic goals, patient tolerance, and individual risk profiles.

For patients with ASCVD, exercise should be regarded as a long-term non-pharmacological component of comprehensive disease management rather than merely an adjunct to medication. Therefore, clinical decision-making should not focus on identifying a single universally optimal exercise modality. Instead, clinicians should first define the primary therapeutic goal, such as improving exercise tolerance or metabolic status, and then select an appropriate intervention by considering both comparative evidence and practical feasibility.

These findings may inform the development of goal-oriented and individualized exercise prescriptions for patients with ASCVD. Future research should focus on optimizing exercise type, intensity, duration, and adherence. Additional high-quality head-to-head trials with longer follow-up are needed to confirm the comparative effectiveness of different exercise modalities and support more standardized exercise prescriptions.

## Data Availability

The original contributions presented in the study are included in the article/**Supplementary Material.** Further inquiries can be directed to the corresponding authors.
