# Supplementary material for: Exercise patterns and atherosclerosis: a network meta-analysis based on randomized controlled trials
Source: Front Physiol. 2026 Aug 18;17:1911464. doi: 10.3389/fphys.2026.1911464 (PMC13529447; doi:10.3389/fphys.2026.1911464)
Supplement: Supplementary file 1 [file DataSheet1.docx]

**SUPPLEMENTARY MATERIAL**

**Table of Contents**

**Supplementary Methods 1:** **Database search strategy.**

**Supplementary Methods 2: Conversion formula for standard.**

**Supplementary Table S1: Specific inclusion and exclusion criteria for this study**

**Supplementary Table S2: Basic definitions of each indicator.**

# Supplementary Table S3: Demographic characteristics of included studies.

# Supplementary Table S4: Details of interventions conducted in the trial.

**Supplementary Table S5: Exercise Therapy Classification.**

# Supplementary Table S6: Results of pairwise comparisons between groups for 6MWD.

**Supplementary Table S7: The SUCRA Values of 6MWD.**

# Supplementary Table S8: Results of pairwise comparisons between groups for PWV.

**Supplementary Table S9: The SUCRA Values of PWV.**

# Supplementary Table S10: Results of pairwise comparisons between groups for TC.

**Supplementary Table S11: The SUCRA Values of TC.**

# Supplementary Table S12: Results of pairwise comparisons between groups for TG.

**Supplementary Table S13: The SUCRA Values of TG.**

# Supplementary Table S14: Results of pairwise comparisons between groups for HDL-C.

**Supplementary Table S15: The SUCRA Values of HDL-C.**

# Supplementary Table S16: Results of pairwise comparisons between groups for LDL-C.

**Supplementary Table S17: The SUCRA Values of LDL-C.**

**Supplementary Figure S1: Risk of bias assessment included in the study.**

**Supplementary Figure S2: Results of pairwise comparisons between groups for 6MWD.**

**Supplementary Figure S3:** **Pooled Forest Plot for 6MWD scores, AE and CT, RT and CT, SAE and CT, as well as SRE and CT.**

**Supplementary Figure S4: Paired meta-sensitivity analysis of 6MWD**

**Supplementary Figure S5: Results of pairwise comparisons between groups for the PWV**

**Supplementary Figure S6:** **Pooled Forest Plot for PWV scores, AE and CT, AERT and CT, IHE and CT, QD and CT, as well as TCC and CT.**

**Supplementary Figure S7: Paired meta-sensitivity analysis of PWV.**

**Supplementary Figure S8: Results of pairwise comparisons between groups for the TC.**

**Supplementary Figure S9:** **Pooled Forest Plot for TC scores, AE and CT, AERT and CT, BFRT and CT, QD and CT, RT and CT, as well as TCC and CT.**

**Supplementary Figure S10: Paired meta-sensitivity analysis of TC.**

**Supplementary Figure S11: Results of pairwise comparisons between groups for the TG.**

**Supplementary Figure S12:** **Pooled Forest Plot for TG scores, AE and CT, BFRT and CT, QD and CT, RT and CT,as well as TCC and CT.**

**Supplementary Figure S13: Paired meta-sensitivity analysis of TG.**

**Supplementary Figure S14: Results of pairwise comparisons between groups for the HDL-C.**

**Supplementary Figure S15:** **Pooled Forest Plot for HDL-C scores, AE and CT, AERT and CT, QD and CT, RT and CT,as well as TCC and CT.**

**Supplementary Figure S16: Paired meta-sensitivity analysis of HDL-C.**

**Supplementary Figure S17: Results of pairwise comparisons between groups for the LDL-C.**

**Supplementary Figure S18: Pooled** **Forest Plot for LDL-C scores, AE and CT, AERT and CT, BFRT and CT, RT and CT,as well as TCC and CT.**

**Supplementary Figure S19: Paired meta-sensitivity analysis of LDL-C.**

**Supplementary Methods 1: Database search strategy.**

**Web of science**

#1

TS=((Exercise Therapy OR Rehabilitation Exercise OR Exercise, Rehabilitation OR Exercises, Rehabilitation OR Rehabilitation Exercises OR Therapy, Exercise OR Exercise Therapies OR Therapies, Exercise OR Remedial Exercise OR Exercise, Remedial OR Exercises, Remedial OR Remedial Exercises) OR (Muscle Stretching Exercises OR Exercise, Muscle Stretching OR Muscle Stretching Exercise OR Active Stretching OR Stretching, Active OR Static-Active Stretching OR Static Active Stretching OR Stretching, Static-Active OR Proprioceptive Neuromuscular Facilitation (PNF) Stretching OR Proprioceptive Neuromuscular Facilitation OR Neuromuscular Facilitation, Proprioceptive OR Proprioceptive Neuromuscular Facilitations OR PNF Stretching OR PNF Stretchings OR Stretching, PNF OR PNF Stretching Exercise OR Exercise, PNF Stretching OR PNF Stretching Exercises OR Stretching Exercise, PNF OR Passive Stretching OR Stretching, Passive OR Static-Passive Stretching OR Static Passive Stretching OR Stretching, Static-Passive OR Relaxed Stretching OR Stretching, Relaxed OR Static Stretching OR Stretching, Static OR Isometric Stretching OR Stretching, Isometric OR Dynamic Stretching OR Stretching, Dynamic OR Ballistic Stretching OR Stretching, Ballistic) OR (Aquatic Therapy OR Hydrotherapy OR Therapy, Aquatic OR Pool Therapy OR Therapy, Pool OR Ai Chi Therapy OR Therapies, Ai Chi OR Therapy, Ai Chi OR Water Tai Chi Therapy OR Aquatic Exercise Therapy OR Exercise Therapy, Aquatic OR Therapy, Aquatic Exercise OR Water Exercise Therapy OR Exercise Therapy, Water OR Therapy, Water Exercise OR Water-based Exercise OR Aqua) OR (Resistance Training OR Training, Resistance OR Strength Training OR Training, Strength OR Weight-Lifting Strengthening Program OR Strengthening Programs, Weight-Lifting OR Strengthening Program, Weight-Lifting OR Weight Lifting Strengthening Program OR Weight-Lifting Strengthening Programs OR Weight-Lifting Exercise Program OR Exercise Programs, Weight-Lifting OR Exercise Program, Weight-Lifting OR Weight Lifting Exercise Program OR Weight-Lifting Exercise Programs OR Weight-Bearing Strengthening Program OR Strengthening Programs, Weight-Bearing OR Strengthening Program, Weight-Bearing OR Weight bearing Strengthening Program OR Weight-Bearing Strengthening Programs OR Weight-Bearing Exercise Program OR Exercise Programs, Weight-Bearing OR Exercise Program, Weight-Bearing OR Weight bearing Exercise Program OR Weight-Bearing Exercise Programs OR Muscle Strength Training) OR ("Yoga" OR "Tai Ji" OR Tai-ji OR Tai Chi OR Chi, Tai OR Tai Ji Quan OR Ji Quan, Tai OR Quan, Tai Ji OR Taiji OR Taijiquan OR "T'ai Chi" OR Tai Chi Chuan OR "Qigong" OR Qi Gong OR Ch'i Kung OR "Exercise Movement Techniques" OR Movement Techniques, Exercise OR Exercise Movement Technics OR Pilates-Based Exercises OR Exercises, Pilates-Based OR Pilates Based Exercises OR Pilates Training OR Training, Pilates OR McKenzie method OR Heckscher training OR running OR jogging OR exergame OR Swiss balls) OR ("Breathing Exercises" OR Inspiratory muscle training OR Expiratory muscle training OR Exercise, Breathing OR Respiratory Muscle Training OR Muscle Training, Respiratory OR Training, Respiratory Muscle) OR ("Exercise" OR "High-Intensity Interval Training" OR High Intensity Interval Training OR High-Intensity Interval Trainings OR Interval Training, High-Intensity OR Interval Trainings, High-Intensity OR Training) OR (balance training OR balance trainings OR balance exercise OR balance exercises OR flexibility training OR flexibility trainings OR flexibility exercise OR flexibility exercises OR joint mobility training OR joint mobility trainings OR joint mobility exercise OR joint mobility exercises OR functional movement training OR functional movement trainings OR functional movement exercise OR functional movement exercises OR stability training OR stability trainings OR stability exercise OR stability exercises OR medical gymnastics))

#2

TS=(randomized controlled trial OR controlled clinical trial OR randomized OR placebo OR clinical trials as topic OR RCT)

#3

TS=(Atheroscleroses OR Atherogenesis OR Atherogeneses)

**Cochrane**

#1

((Exercise Therapy OR Rehabilitation Exercise OR Exercise, Rehabilitation OR Exercises, Rehabilitation OR Rehabilitation Exercises OR Therapy, Exercise OR Exercise Therapies OR Therapies, Exercise OR Remedial Exercise OR Exercise, Remedial OR Exercises, Remedial OR Remedial Exercises) OR (Muscle Stretching Exercises OR Exercise, Muscle Stretching OR Muscle Stretching Exercise OR Active Stretching OR Stretching, Active OR Static - Active Stretching OR Static Active Stretching OR Stretching, Static - Active OR Proprioceptive Neuromuscular Facilitation (PNF) Stretching OR Proprioceptive Neuromuscular Facilitation OR Neuromuscular Facilitation, Proprioceptive OR Proprioceptive Neuromuscular Facilitations OR PNF Stretching OR PNF Stretchings OR Stretching, PNF OR PNF Stretching Exercise OR Exercise, PNF Stretching OR PNF Stretching Exercises OR Stretching Exercise, PNF OR Passive Stretching OR Stretching, Passive OR Static - Passive Stretching OR Static Passive Stretching OR Stretching, Static - Passive OR Relaxed Stretching OR Stretching, Relaxed OR Static Stretching OR Stretching, Static OR Isometric Stretching OR Stretching, Isometric OR Dynamic Stretching OR Stretching, Dynamic OR Ballistic Stretching OR Stretching, Ballistic) OR (Aquatic Therapy OR Hydrotherapy OR Therapy, Aquatic OR Pool Therapy OR Therapy, Pool OR Ai Chi Therapy OR Therapies, Ai Chi OR Therapy, Ai Chi OR Water Tai Chi Therapy OR Aquatic Exercise Therapy OR Exercise Therapy, Aquatic OR Therapy, Aquatic Exercise OR Water Exercise Therapy OR Exercise Therapy, Water OR Therapy, Water Exercise OR Water - based Exercise OR Aqua) OR (Resistance Training OR Training, Resistance OR Strength Training OR Training, Strength OR Weight - Lifting Strengthening Program OR Strengthening Programs, Weight - Lifting OR Strengthening Program, Weight - Lifting OR Weight Lifting Strengthening Program OR Weight - Lifting Strengthening Programs OR Weight - Lifting Exercise Program OR Exercise Programs, Weight - Lifting OR Exercise Program, Weight - Lifting OR Weight Lifting Exercise Program OR Weight - Lifting Exercise Programs OR Weight - Bearing Strengthening Program OR Strengthening Programs, Weight - Bearing OR Strengthening Program, Weight - Bearing OR Weight bearing Strengthening Program OR Weight - Bearing Strengthening Programs OR Weight - Bearing Exercise Program OR Exercise Programs, Weight - Bearing OR Exercise Program, Weight - Bearing OR Weight bearing Exercise Program OR Weight - Bearing Exercise Programs OR Muscle Strength Training) OR ("Yoga" OR "Tai Ji" OR Tai - ji OR Tai Chi OR Chi, Tai OR Tai Ji Quan OR Ji Quan, Tai OR Quan, Tai Ji OR Taiji OR Taijiquan OR "T'ai Chi" OR Tai Chi Chuan OR "Qigong" OR Qi Gong OR Ch'i Kung OR "Exercise Movement Techniques" OR Movement Techniques, Exercise OR Exercise Movement Technics OR Pilates - Based Exercises OR Exercises, Pilates - Based OR Pilates Based Exercises OR Pilates Training OR Training, Pilates OR McKenzie method OR Heckscher training OR running OR jogging OR exergame OR Swiss balls) OR ("Breathing Exercises" OR Inspiratory muscle training OR Expiratory muscle training OR Exercise, Breathing OR Respiratory Muscle Training OR Muscle Training, Respiratory OR Training, Respiratory Muscle) OR ("Exercise" OR "High - Intensity Interval Training" OR High Intensity Interval Training OR High - Intensity Interval Trainings OR Interval Training, High - Intensity OR Interval Trainings, High - Intensity OR Training) OR (balance training OR balance trainings OR balance exercise OR balance exercises OR flexibility training OR flexibility trainings OR flexibility exercise OR flexibility exercises OR joint mobility training OR joint mobility trainings OR joint mobility exercise OR joint mobility exercises OR functional movement training OR functional movement trainings OR functional movement exercise OR functional movement exercises OR stability training OR stability trainings OR stability exercise OR stability exercises OR medical gymnastics)) AND (randomized controlled trial OR controlled clinical trial OR randomized OR placebo OR clinical trials as topic OR RCT)

#2

（Atheroscleroses OR Atherogenesis OR Atherogeneses）

**Embase**

#1

("rehabilitation exercise*":ab,ti,kw OR "rehabilitation exercises":ab,ti,kw OR "exercises, rehabilitation":ab,ti,kw OR "exercises rehabilitation":ab,ti,kw OR "rehabilitation therapies":ab,ti,kw OR "therapies, exercise":ab,ti,kw OR "exercise therapy*":ab,ti,kw OR "exercise therapies":ab,ti,kw OR "remedial exercise*":ab,ti,kw OR "remedial exercises":ab,ti,kw OR "exercise, remedial":ab,ti,kw OR "exercises, remedial":ab,ti,kw OR "muscle stretching exercise*":ab,ti,kw OR "muscle stretching exercises":ab,ti,kw OR "exercise, muscle stretching":ab,ti,kw OR "active stretching":ab,ti,kw OR "stretching, active":ab,ti,kw OR "static-active stretching":ab,ti,kw OR "static active stretching":ab,ti,kw OR "stretching, static-active":ab,ti,kw OR "proprioceptive neuromuscular facilitation (PNF) stretching":ab,ti,kw OR "proprioceptive neuromuscular facilitation":ab,ti,kw OR "neuromuscular facilitation, proprioceptive":ab,ti,kw OR "proprioceptive neuromuscular facilitations":ab,ti,kw OR "PNF stretching":ab,ti,kw OR "PNF stretchings":ab,ti,kw OR "stretching, PNF":ab,ti,kw OR "PNF stretching exercise":ab,ti,kw OR "exercise, PNF stretching":ab,ti,kw OR "PNF stretching exercises":ab,ti,kw OR "stretching exercise, PNF":ab,ti,kw OR "passive stretching":ab,ti,kw OR "stretching, passive":ab,ti,kw OR "static-passive stretching":ab,ti,kw OR "static passive stretching":ab,ti,kw OR "stretching, static-passive":ab,ti,kw OR "relaxed stretching":ab,ti,kw OR "stretching, relaxed":ab,ti,kw OR "static stretching":ab,ti,kw OR "stretching, static":ab,ti,kw OR "isometric stretching":ab,ti,kw OR "stretching, isometric":ab,ti,kw OR "dynamic stretching":ab,ti,kw OR "stretching, dynamic":ab,ti,kw OR "ballistic stretching":ab,ti,kw OR "stretching, ballistic":ab,ti,kw OR "aquatic therapy":ab,ti,kw OR "hydrotherapy":ab,ti,kw OR "therapy, aquatic":ab,ti,kw OR "pool therapy":ab,ti,kw OR "therapy, pool":ab,ti,kw OR "ai chi therapy":ab,ti,kw OR "therapies, ai chi":ab,ti,kw OR "therapy, ai chi":ab,ti,kw OR "water tai chi therapy":ab,ti,kw OR "aquatic exercise therapy":ab,ti,kw OR "exercise therapy, aquatic":ab,ti,kw OR "therapy, aquatic exercise":ab,ti,kw OR "water exercise therapy":ab,ti,kw OR "exercise therapy, water":ab,ti,kw OR "therapy, water exercise":ab,ti,kw OR "water-based exercise":ab,ti,kw OR "aqua":ab,ti,kw OR "resistance training":ab,ti,kw OR "training, resistance":ab,ti,kw OR "strength training":ab,ti,kw OR "training, strength":ab,ti,kw OR "weight-lifting strengthening program*":ab,ti,kw OR "strengthening programs, weight-lifting":ab,ti,kw OR "strengthening program, weight-lifting":ab,ti,kw OR "weight lifting strengthening program":ab,ti,kw OR "weight-lifting strengthening programs":ab,ti,kw OR "weight-lifting exercise program*":ab,ti,kw OR "exercise programs, weight-lifting":ab,ti,kw OR "exercise program, weight-lifting":ab,ti,kw OR "weight lifting exercise program":ab,ti,kw OR "weight-lifting exercise programs":ab,ti,kw OR "weight-bearing strengthening program*":ab,ti,kw OR "strengthening programs, weight-bearing":ab,ti,kw OR "strengthening program, weight-bearing":ab,ti,kw OR "weight bearing strengthening program":ab,ti,kw OR "weight-bearing strengthening programs":ab,ti,kw OR "weight-bearing exercise program*":ab,ti,kw OR "exercise programs, weight-bearing":ab,ti,kw OR "exercise program, weight-bearing":ab,ti,kw OR "weight bearing exercise program":ab,ti,kw OR "weight-bearing exercise programs":ab,ti,kw OR "muscle strength training":ab,ti,kw OR "yoga":ab,ti,kw OR "tai ji":ab,ti,kw OR "tai-ji":ab,ti,kw OR "tai chi":ab,ti,kw OR "chi, tai":ab,ti,kw OR "tai ji quan":ab,ti,kw OR "ji quan, tai":ab,ti,kw OR "quan, tai ji":ab,ti,kw OR "taiji":ab,ti,kw OR "taijiquan":ab,ti,kw OR "tai chi":ab,ti,kw OR "tai chi chuan":ab,ti,kw OR "qigong":ab,ti,kw OR "qi gong":ab,ti,kw OR "chi kung":ab,ti,kw OR "exercise movement techniques":ab,ti,kw OR "movement techniques, exercise":ab,ti,kw OR "exercise movement technics":ab,ti,kw OR "pilates-based exercises":ab,ti,kw OR "exercises, pilates-based":ab,ti,kw OR "pilates based exercises":ab,ti,kw OR "pilates training":ab,ti,kw OR "training, pilates":ab,ti,kw OR "mckenzie method":ab,ti,kw OR "heckscher training":ab,ti,kw OR "running":ab,ti,kw OR "jogging":ab,ti,kw OR "exergame":ab,ti,kw OR "swiss balls":ab,ti,kw OR "breathing exercises":ab,ti,kw OR "inspiratory muscle training":ab,ti,kw OR "expiratory muscle training":ab,ti,kw OR "exercise, breathing":ab,ti,kw OR "respiratory muscle training":ab,ti,kw OR "muscle training, respiratory":ab,ti,kw OR "training, respiratory muscle":ab,ti,kw OR "exercise":ab,ti,kw OR "high-intensity interval training":ab,ti,kw OR "high intensity interval training":ab,ti,kw OR "high-intensity interval trainings":ab,ti,kw OR "interval training, high-intensity":ab,ti,kw OR "interval trainings, high-intensity":ab,ti,kw OR "training":ab,ti,kw OR "balance training":ab,ti,kw OR "balance trainings":ab,ti,kw OR "balance exercise":ab,ti,kw OR "balance exercises":ab,ti,kw OR "flexibility training":ab,ti,kw OR "flexibility trainings":ab,ti,kw OR "flexibility exercise":ab,ti,kw OR "flexibility exercises":ab,ti,kw OR "joint mobility training":ab,ti,kw OR "joint mobility trainings":ab,ti,kw OR "joint mobility exercise":ab,ti,kw OR "joint mobility exercises":ab,ti,kw OR "functional movement training":ab,ti,kw OR "functional movement trainings":ab,ti,kw OR "functional movement exercise":ab,ti,kw OR "functional movement exercises":ab,ti,kw OR "stability training":ab,ti,kw OR "stability trainings":ab,ti,kw OR "stability exercise":ab,ti,kw OR "stability exercises":ab,ti,kw OR "medical gymnastics":ab,ti,kw) AND (randomized*:ab,ti,kw OR controlled clinical trial*:ab,ti,kw OR random*:ab,ti,kw OR placebo*:ab,ti,kw OR RCT:ab,ti,kw) AND (Atheroscleroses*:ab,ti,kw OR Atherogenesis*:ab,ti,kw OR Atherogeneses*:ab,ti,kw)

#2

randomized*:ab,ti,kw OR controlled clinical trial*:ab,ti,kw OR random*:ab,ti,kw OR placebo*:ab,ti,kw OR RCT:ab,ti,kw

#3

Atheroscleroses*:ab,ti,kw OR Atherogenesis*:ab,ti,kw OR Atherogeneses*:ab,ti,kw

**PubMed**

#1

((Exercise Therapy OR Rehabilitation Exercise OR Exercise, Rehabilitation OR Exercises, Rehabilitation OR Rehabilitation Exercises OR Therapy, Exercise OR Exercise Therapies OR Therapies, Exercise OR Remedial Exercise OR Exercise, Remedial OR Exercises, Remedial OR Remedial Exercises) OR (Muscle Stretching Exercises OR Exercise, Muscle Stretching OR Muscle Stretching Exercise OR Active Stretching OR Stretching, Active OR Static-Active Stretching OR Static Active Stretching OR Stretching, Static-Active OR Proprioceptive Neuromuscular Facilitation (PNF) Stretching OR Proprioceptive Neuromuscular Facilitation OR Neuromuscular Facilitation, Proprioceptive OR Proprioceptive Neuromuscular Facilitations OR PNF Stretching OR PNF Stretchings OR Stretching, PNF OR PNF Stretching Exercise OR Exercise, PNF Stretching OR PNF Stretching Exercises OR Stretching Exercise, PNF OR Passive Stretching OR Stretching, Passive OR Static-Passive Stretching OR Static Passive Stretching OR Stretching, Static-Passive OR Relaxed Stretching OR Stretching, Relaxed OR Static Stretching OR Stretching, Static OR Isometric Stretching OR Stretching, Isometric OR Dynamic Stretching OR Stretching, Dynamic OR Ballistic Stretching OR Stretching, Ballistic) OR (Aquatic Therapy OR Hydrotherapy OR Therapy, Aquatic OR Pool Therapy OR Therapy, Pool OR Ai Chi Therapy OR Therapies, Ai Chi OR Therapy, Ai Chi OR Water Tai Chi Therapy OR Aquatic Exercise Therapy OR Exercise Therapy, Aquatic OR Therapy, Aquatic Exercise OR Water Exercise Therapy OR Exercise Therapy, Water OR Therapy, Water Exercise OR Water-based Exercise OR Aqua) OR (Resistance Training OR Training, Resistance OR Strength Training OR Training, Strength OR Weight-Lifting Strengthening Program OR Strengthening Programs, Weight-Lifting OR Strengthening Program, Weight-Lifting OR Weight Lifting Strengthening Program OR Weight-Lifting Strengthening Programs OR Weight-Lifting Exercise Program OR Exercise Programs, Weight-Lifting OR Exercise Program, Weight-Lifting OR Weight Lifting Exercise Program OR Weight-Lifting Exercise Programs OR Weight-Bearing Strengthening Program OR Strengthening Programs, Weight-Bearing OR Strengthening Program, Weight-Bearing OR Weight bearing Strengthening Program OR Weight-Bearing Strengthening Programs OR Weight-Bearing Exercise Program OR Exercise Programs, Weight-Bearing OR Exercise Program, Weight-Bearing OR Weight bearing Exercise Program OR Weight-Bearing Exercise Programs OR Muscle Strength Training) OR ("Yoga" OR "Tai Ji" OR Tai-ji OR Tai Chi OR Chi, Tai OR Tai Ji Quan OR Ji Quan, Tai OR Quan, Tai Ji OR Taiji OR Taijiquan OR "T'ai Chi" OR Tai Chi Chuan OR "Qigong" OR Qi Gong OR Ch'i Kung OR "Exercise Movement Techniques" OR Movement Techniques, Exercise OR Exercise Movement Technics OR Pilates-Based Exercises OR Exercises, Pilates-Based OR Pilates Based Exercises OR Pilates Training OR Training, Pilates OR McKenzie method OR Heckscher training OR running OR jogging OR exergame OR Swiss balls) OR ("Breathing Exercises" OR Inspiratory muscle training OR Expiratory muscle training OR Exercise, Breathing OR Respiratory Muscle Training OR Muscle Training, Respiratory OR Training, Respiratory Muscle) OR ("Exercise" OR "High-Intensity Interval Training" OR High Intensity Interval Training OR High-Intensity Interval Trainings OR Interval Training, High-Intensity OR Interval Trainings, High-Intensity OR Training) OR (balance training OR balance trainings OR balance exercise OR balance exercises OR flexibility training OR flexibility trainings OR flexibility exercise OR flexibility exercises OR joint mobility training OR joint mobility trainings OR joint mobility exercise OR joint mobility exercises OR functional movement training OR functional movement trainings OR functional movement exercise OR functional movement exercises OR stability training OR stability trainings OR stability exercise OR stability exercises OR medical gymnastics))

#2

(randomized controlled trial OR controlled clinical trial OR randomized OR placebo OR clinical trials as topic OR RCT)

#3

(Atheroscleroses OR Atherogenesis OR Atherogeneses)

**Supplementary Methods 2: Conversion formula for standard deviation (SD).**

**Standard Error to Standard Deviation:**

SD=SE×$\sqrt{\text{N}}$ where SE is the standard error and N is the sample size.

**95% Confidence Interval to Standard Deviation:**

1.For sample sizes in the test and control groups that are greater than or equal to 100, the standard deviation (SD) can be calculated as follows: SD = (Upper limit of credible intervals - Lower limit of credible intervals) / 3.92.

2.For sample sizes in the test and control groups that are less than or equal to 60, the standard deviation (SD) is determined by the formula: SD = (Upper limit of credible intervals - Lower limit of credible intervals) / tinv(1-0.95, n-1). 3. For studies where the sample sizes in each group range between 60 and 100, either of the aforementioned methods may be employed.

**Combined Subgroup**

In the combined subgroup analysis, let the sample size of subgroup A be denoted as N_1_, with a mean of M_1_ and a standard deviation of SD_1_. For subgroup B, let the sample size be N_2_, with a mean of M_2_ and a standard deviation of SD_2_. The total combined sample size is (N = N_1_ + N_2_). The combined mean M is calculated as (M = N_1_M_1_+ N_2_M_2_)/(N_1_ + N_2_).

# Supplementary Table S1: Specific inclusion and exclusion criteria for this study

| PICOS Elements | Inclusion Criteria | Exclusion Criteria |
| --- | --- | --- |
| P (Participants) | Adults (≥ 18 years) with clinically diagnosed ASCVD, according to the 2019 ESC/EAS Guidelines for the Management of Dyslipidemias: Lipid Modification to Reduce Cardiovascular Risk or explicit clinical criteria reported in the original studies. | Animal studies; non-ASCVD populations; unclear diagnostic criteria; participants < 18 years. |
| I (Interventions) | The experimental group will undergo at least one exercise intervention, including but not limited to AE, RT, AERT, TCC, QD, SAE, SRE, BFRT, HTAE, and SE; exercise interventions may be implemented in addition to standard ASCVD treatment measures, while continuing standard ASCVD treatment. | Studies that did not specify a clear exercise intervention, for which the type or protocol of exercise could not be determined, or that involved only a single non-exercise intervention (such as medication, surgery, or diet) and from which the effects of exercise could not be extracted. |
| C (Comparators) | The control group received standard ASCVD treatment measures, including conventional therapy, health education, general care, waiting lists, placebo interventions, or other unstructured exercise interventions; studies directly comparing different types of exercise were also included. | Studies that do not include an appropriate control group, or in which the control measures cannot be clearly identified or cannot be effectively compared with the exercise intervention in the experimental group. |
| O (Outcomes) | Report at least one of the following outcome measures: 6MWD, PWV, TC, TG, HDL-C, or LDL-C. | Studies that did not report any of the aforementioned outcome measures; articles in which data were presented in graphical form and could not be directly analyzed; or articles from which valid data could not be obtained or extracted. |
| S（Study design） | RCTs; studies must be in English. | Studies that have been previously published or contain duplicated information; non-randomized controlled trials; conference reports, letters, case reports, and articles for which the full text is unavailable. |

**Supplementary Table S2 Basic definitions of each indicator.**

| **Mean difference (MD)** | The MD serves as an effect size metric employed to quantify the absolute mean difference between two independent data groups. By preserving the original units of measurement for a given continuous outcome, it maintains the direct clinical interpretability of the data, thereby facilitating a straightforward assessment of the intervention's true magnitude. This reliance on natural units enables the accurate pooling of data, allowing for the direct synthesis of studies exclusively when they utilize the identical measurement scale or diagnostic tool. |
| --- | --- |
| **95% credible interval (CI)** | In the context of frequentism metwork meta-analysis, the 95% confidence interval (CI) serves as a fundamental tool for evaluating the uncertainty associated with the effects of interventions. This approach is grounded in classical statistical theory, which posits that the effect size is a fixed parameter. The CI quantifies the "true effect" by synthesizing both direct and indirect comparison evidence, thereby delineating the potential range of effect sizes. Its application spans several critical scenarios: it can quantify the impact of intervention measures (such as risk ratios and mean differences) and determine statistical significance by assessing whether the CI includes null values (for instance, whether the CI encompasses 0 for continuous variables or 1 for binary variables). Furthermore, the CI can assess the reliability of evidence by examining the extent of overlap between direct and indirect evidence in consistency tests. Unlike the credible interval used in Bayesian network meta-analysis (NMA), the 95% CI in this context is fundamentally an inference about true values under repeated sampling, emphasizing objective evidence inference. It provides a rigorous statistical framework for integrating complex evidence networks and supports evidence-based decision-making. |
| **Statistical difference** | In the context of frequentism metwork meta-analysis, the determination of statistical significance is grounded in classical statistical theory. The primary objective is to assess whether the observed differences between interventions are "non-random" by examining the effect size and its corresponding 95% confidence interval (CI). For relative effect measures, such as the hazard ratio (HR) or odds ratio (OR), a 95% CI that does not encompass the null value of 1 indicates a statistically significant difference. Similarly, for absolute effect measures, such as the mean difference (MD) or standardized mean difference (SMD), a 95% CI that does not include 0 signifies statistical significance. This evaluative criterion is applied consistently, regardless of whether the comparison is a direct head-to-head test or an indirect comparison facilitated through an intermediate intervention. |
| **Heterogeneity** | In the context of meta-analysis, heterogeneity pertains to the variability among included studies, which can manifest across various dimensions such as study outcomes, methodologies, population characteristics, or intervention strategies. This variability fundamentally indicates that the effect size of each individual study cannot be adequately accounted for by a single common effect. The concept of heterogeneity encompasses statistical, clinical, and methodological dimensions. Statistical heterogeneity is assessed by quantifying the degree of variation in effect sizes, with the I² statistic frequently employed as a measure; for instance, an I² value exceeding 50% is indicative of moderate to high heterogeneity. |
| **SUCRA (Surface Under the Cumulative RANK Curve)** | In the context of NMA, SUCRA (Surface Under the Cumulative RANK Curve) serves as a metric to assess the likelihood of ranking the relative efficacy of various interventions. This measure effectively consolidates the ranking probabilities of different interventions into a single value ranging from 0 to 1, facilitating intuitive comparison and ranking. A SUCRA value approaching 1 suggests that an intervention is more likely to be effective, whereas a value nearing 0 indicates a higher likelihood of being the least effective treatment. By weighting the cumulative probabilities of all potential ranking positions, SUCRA synthesizes evidence from both direct and indirect comparisons, thereby overcoming the limitations associated with rankings based solely on point estimates. Additionally, the 95% confidence interval provides insight into the stability of these rankings. As a non-parametric measure, SUCRA does not require assumptions regarding the distribution of effect sizes. In the context of network comparisons involving multiple interventions, SUCRA offers a quantitative basis for clinical decision-making, aiding researchers and policymakers in identifying the most favorable interventions. |

# Supplementary Table S3 Demographic characteristics of included studies.

# This supplementary file presents the demographic characteristics and intervention details of the included studies. Each study is arranged in a long-table format, with the first row representing the control or comparator group and the second row representing the experimental or intervention group. The study column indicates the first author and year of publication. The sample size, age, BMI, disease duration, frequency, session time, and exact dose were extracted separately for the control and experimental groups when available. The intervention duration indicates the formal intervention period in weeks, excluding follow-up or detraining periods. The exact dose column describes the detailed exercise prescription, including exercise mode, intensity, duration, progression, sets or repetitions, and supervision when reported. NR: not reported; 6MWD: six-minute walking distance; PWV: pulse wave velocity; TC: total cholesterol; TG: triglycerides; HDL-C: high-density lipoprotein cholesterol; LDL-C: low-density lipoprotein cholesterol; BMI: body mass index; PAD: peripheral artery disease; T2DM: type 2 diabetes mellitus; AE: aerobic exercise; RE: resistance exercise; BFR: blood flow restriction; BFR-RT: blood flow-restrictive resistance training; HEP: home exercise program; SET: supervised exercise therapy; LBET: land-based exercise therapy; HWET: heated water-based exercise therapy; IHT: isometric handgrip training; CET: combined exercise training; ABI: ankle-brachial index; HRR: heart rate reserve; RPE: rating of perceived exertion; SEM: standard error of the mean; IQR: interquartile range.

| **Study** | **Group** | **Group / comparator name** | **Sample size** | **Age** | **BMI** | **Intervention duration** | **Frequency** | **Time/ session** | **Disease duration** | **Region** | **Treatment / population** | **Outcome** |
| --- | --- | --- | --- | --- | --- | --- | --- | --- | --- | --- | --- | --- |
| Ratajczak 2019 | Control group | Endurance–strength training | n=17 | 49±10 years | 35.0±3.9 kg/m² | 12 | 3 sessions/week; 36 sessions total | 60 min/session | NR | Poznan, Poland | Women with obesity; exercise training | ③；④；⑤；⑥ |
|  | Experimental group | Endurance training | n=22 | 51±8 years | 35.9±5.2 kg/m² | 12 | 3 sessions/week; 36 sessions total | 60 min/session | NR | Poznan, Poland | Women with obesity; exercise training | ③；④；⑤；⑥ |
| Manfredini 2024 | Control group | C-WA traditional “go home and walk” walking advice | n=34 | 73±7 years | 26±5 kg/m² | 24 | ≥3 days/week | 30 min walking/session | 4±4 years of claudication | Ferrara, Italy | PAD, Leriche-Fontaine stage II; home-based exercise therapy | ① |
|  | Experimental group | TiTo-SHB structured home-based pain-free interval walking | n=34 | 71±7 years | 27±4 kg/m² | 24 | 2 sessions/day, 6 days/week | 16 min/session total, including 8 min walking | 3±4 years of claudication | Ferrara, Italy | PAD, Leriche-Fontaine stage II; home-based exercise therapy | ① |
| Kearney 2014 | Control group | Stretching control | n=25 | 45±7.4 years | 29.14±4.25 kg/m² | 26 | Stretching twice daily, 5 days/week | NR | NR | Northern Ireland, UK | Sedentary overweight adults; lifestyle brisk walking intervention | ②；③；④；⑤；⑥ |
|  | Experimental group | Accumulated brisk walking with telephone support; two walking-support arms combined | n=52 | 45±6.2 years | 29.19±4.32 kg/m² | 26 | 5 days/week | 3×10-min bouts/day; 30 min/day | NR | Northern Ireland, UK | Sedentary overweight adults; lifestyle brisk walking intervention | ②；③；④；⑤；⑥ |
| Son 2025 | Control group | Non-exercise control | n=12 | 32.58±1.73 years | 26.94±1.90 kg/m² | 12 | No exercise intervention | NR | NR | Republic of Korea | Obese adult men; circuit training | ② |
|  | Experimental group | Circuit training | n=13 | 32.15±1.21 years | 26.84±1.57 kg/m² | 12 | 3 sessions/week | Weeks 1–6: 55 min; weeks 7–12: 65 min | NR | Republic of Korea | Obese adult men; circuit training | ② |
| Bronas 2011 | Control group | Usual-care control | n=8 | 70±7.8 years | 29.2±5.4 kg/m² | 12 | Usual care | NR | NR | Minneapolis–St Paul, Minnesota, USA | PAD-related claudication; supervised aerobic exercise | NR |
|  | Experimental group | Supervised arm-ergometry aerobic training; supervised treadmill-walking training | Arm-ergometry n=10; treadmill n=10 | Arm: 64±8.6; treadmill: 63±11.5 years | Arm: 29.9±8.8; treadmill: 26.4±3.3 kg/m² | 12 | 3 sessions/week; 3 h/week supervised exercise | 60 min/session including warm-up and cool-down | NR | Minneapolis–St Paul, Minnesota, USA | PAD-related claudication; supervised aerobic exercise | NR |
| Lin 2021 | Control group | General rehabilitation only | n=20 | 70.5±1.9 SEM years | NR | 12 | General rehabilitation schedule NR | NR | NR; inclusion required PAD duration >2 weeks | Keelung/Taoyuan, Taiwan | PAD; cycling exercise training plus general rehabilitation | ① |
|  | Experimental group | General rehabilitation + cycling exercise training (GR+CET) | n=20 | 71.1±1.5 SEM years | NR | 12 | 3 days/week | 30 min/day | NR; inclusion required PAD duration >2 weeks | Keelung/Taoyuan, Taiwan | PAD; cycling exercise training plus general rehabilitation | ① |
| McDermott 2019 | Control group | Attention control | n=53 | 68.1±12.2 years | 30.5±7.5 kg/m² | 24 | 11 group sessions during months 1–6; telephone contact months 6–12 | Control sessions about 1 h | NR | Chicago, Illinois, USA | PAD; supervised treadmill/resistance exercise | ① |
|  | Experimental group | Supervised treadmill exercise; supervised lower-extremity resistance training | Treadmill n=51; resistance n=52 | Treadmill: 72.0±8.5; resistance: 71.8±10.2 years | Treadmill: 30.2±6.1; resistance: 29.7±6.5 kg/m² | 24 | Exercise groups: 3 sessions/week during months 1–6 | Treadmill progressed from 15 to 40 min/session; resistance session time NR | NR | Chicago, Illinois, USA | PAD; supervised treadmill/resistance exercise | ① |
| McDermott 2025 | Control group | Nonexercise health education control | n=100 | 66.9±7.8 years | 30.4±6.8 kg/m² | 26 | Nonexercise control | NR | NR | Chicago, Illinois, USA | PAD; supervised treadmill exercise | ① |
|  | Experimental group | Supervised treadmill exercise | n=100 | 67.3±9.1 years | 31.0±6.4 kg/m² | 26 | 3 sessions/week | Progressed up to 50 min/session | NR | Chicago, Illinois, USA | PAD; supervised treadmill exercise | ① |
| Jia 2025 | Control group | No non-exercise control; active comparison among 3 training groups | NR | NR | NR | 12 | NR | NR | NR | Jiangsu, China | Young adults; resistance/aerobic/BFR training | ② |
|  | Experimental group | HIRT; HIR-AET; LIR-BFRT | HIRT n=18; HIR-AET n=17; LIR-BFRT n=20 | HIRT: 21.05±0.84; HIR-AET: 20.90±1.02; LIR-BFRT: 21.14±0.58 years | HIRT: 24.68±1.35; HIR-AET: 25.20±1.04; LIR-BFRT: 25.94±0.96 kg/m² | 12 | 3 sessions/week | HIRT about 70 min; HIR-AET about 75–80 min; LIR-BFRT total session duration NR | NR | Jiangsu, China | Young adults; resistance/aerobic/BFR training | ② |
| Bearne 2022 | Control group | Usual care | n=95 | 68.2±9.0 years | 26.9±5.8 kg/m² | 12 | Usual care | NR | NR | 6 hospitals, United Kingdom | Adults with PAD and intermittent claudication | ① |
|  | Experimental group | Home-based walking exercise behavior change intervention | n=95 | 67.6±8.7 years | 26.7±5.7 kg/m² | 12 | Walking goal ≥30 min/day, 3 days/week | Two 60-min in-person sessions + two 20-min telephone sessions; walking goal ≥30 min/day | NR | 6 hospitals, United Kingdom | Adults with PAD and intermittent claudication | ① |
| Oliveira 2015 | Control group | Usual care only | n=42 | 58.5±10.7 years | 26.9 (25.3–29.0) kg/m² | 8 | Usual care | NR | NR; patients 4 weeks after acute MI | Vila Nova de Gaia/Espinho, Portugal | Post-myocardial infarction patients; exercise-based cardiac rehabilitation | ② |
|  | Experimental group | Exercise-based cardiac rehabilitation (ECR) + usual care | n=44 | 55.0±10.7 years | 26.1 (24.5–29.1) kg/m² | 8 | 3 sessions/week | 50 min/session | NR; patients 4 weeks after acute MI | Vila Nova de Gaia/Espinho, Portugal | Post-myocardial infarction patients; exercise-based cardiac rehabilitation | ② |
| McDermott 2021 | Control group | Nonexercise education control | n=65 | 69.5±10.1 years | 30.8±7.3 kg/m² | 52 (12 months) | Weekly educational telephone calls | NR | NR | 4 centers, USA | PAD; low- vs high-intensity home-based walking | ① |
|  | Experimental group | Low-intensity home-based walking exercise; high-intensity home-based walking exercise | Low-intensity n=116; high-intensity n=124 | Low: 69.8±10.1; high: 68.8±8.7 years | Low: 30.3±6.7; high: 31.1±7.3 kg/m² | 52 (12 months) | 5 times/week + weekly coaching calls | Up to 50 min/session | NR | 4 centers, USA | PAD; low- vs high-intensity home-based walking | ① |
| Ohta 2012 | Control group | Control group | n=13 | 71.5±7.4 years | 21.8±2.6 kg/m² | 12 | No exercise intervention | NR | NR | Northern Kyushu, Japan | Post-menopausal elderly women; bench step exercise | ②；③；④；⑤；⑥ |
|  | Experimental group | Home-based bench step exercise | n=13 | 72.2±4.2 years | 23.0±2.6 kg/m² | 12 | 3 times/day | 10–20 min/session; total 140 min/week prescribed | NR | Northern Kyushu, Japan | Post-menopausal elderly women; bench step exercise | ②；③；④；⑤；⑥ |
| Woo 2004 | Control group | Dietary modification only | n=41 | 9.9±0.9 years | 24.5±2.9 kg/m² | 6 weeks + subsequent 1 year exercise follow-up | Diet counseling follow-up only | NR | NR | Hong Kong, China | Overweight/obese children; diet plus exercise vs diet only | ③；④；⑤；⑥ |
|  | Experimental group | Diet + supervised structured exercise program | n=41 | 10.0±1.0 years | 25.4±3.1 kg/m² | 6 weeks + subsequent 1 year exercise follow-up | Twice/week for first 6 weeks, then once weekly for 1 year | 75 min/session | NR | Hong Kong, China | Overweight/obese children; diet plus exercise vs diet only | ③；④；⑤；⑥ |
| Zeng 2024 | Control group | Regular routines / no exercise intervention | n=20 | NR (overall 61.78±5.74 years) | NR | 12 | No exercise intervention | NR | NR | Beijing, China | Middle-aged and elderly women; health Qigong/dance exercise | ②；③；④；⑤ |
|  | Experimental group | Health Qigong combined with Tibetan dance | n=20 | NR (overall 61.78±5.74 years) | NR | 12 | 3 sessions/week | 60 min/session | NR | Beijing, China | Middle-aged and elderly women; health Qigong/dance exercise | ②；③；④；⑤ |
| Park 2020 | Control group | Land-based exercise therapy (LBET; active comparator) | n=25 | 60.0±10.0 years | 22.5±2.4 kg/m² | 12 | 4 sessions/week | 60 min/session | NR | Omaha, Nebraska, USA | Individuals with peripheral artery disease; heated-water vs land-based exercise | ①；② |
|  | Experimental group | Heated-water exercise therapy (HWET) | n=28 | 60.0±9.0 years | 22.8±2.8 kg/m² | 12 | 4 sessions/week | 60 min/session | NR | Omaha, Nebraska, USA | Individuals with peripheral artery disease; heated-water vs land-based exercise | ①；② |
| Correia 2020 | Control group | Sham handgrip/compression-ball control | n=52 | 67±11 years | 26±7 kg/m² | 8 | 3 sessions/week | NR | NR | São Paulo, Brazil | Patients with peripheral artery disease; isometric handgrip training | ② |
|  | Experimental group | Isometric handgrip training (IHT) | n=50 | 66±12 years | 27±5 kg/m² | 8 | 3 sessions/week | ≈20 min/session (4×2-min contractions + 4-min intervals) | NR | São Paulo, Brazil | Patients with peripheral artery disease; isometric handgrip training | ② |
| Laslovich 2020 | Control group | Attention control | n=19 | 68±10.6 years | 28.8±5.2 kg/m² | 12 | Bimonthly online PAD education videos | NR | NR | San Diego, California, USA | Sedentary adults with asymptomatic peripheral arterial disease | ① |
|  | Experimental group | Physical activity sedentary reduction (PASR) lifestyle program | n=19 | 68±7.5 years | 29.5±4.1 kg/m² | 12 | Daily self-monitoring; ≥2 continuous 10-min walking bouts/day targeted | ≥2×10-min walking bouts/day targeted | NR | San Diego, California, USA | Sedentary adults with asymptomatic peripheral arterial disease | ① |
| Gardner 2023 | Control group | Control: light resistance training for 3 months, then walking advice | n=34 | 66±9 years | 31.1±6.1 kg/m² | 78 (18 months) | First 3 months supervised light resistance; then walking advice only | NR | NR | Oklahoma City, Oklahoma, USA | Symptomatic peripheral artery disease with claudication | ① |
|  | Experimental group | Long-term home exercise program (HEP); SET/HEP transition program | HEP n=18; SET/HEP n=20 | HEP: 64±11; SET/HEP: 65±9 years | HEP: 27.1±5.0; SET/HEP: 29.8±4.8 kg/m² | 78 (18 months) | HEP ≥3 days/week; SET/HEP 3 supervised sessions/week initially, then HEP | HEP: 42.7±21.5 min/session; SET/HEP: 30.6±5.4 min/session | NR | Oklahoma City, Oklahoma, USA | Symptomatic peripheral artery disease with claudication | ① |
| Parmenter 2013 | Control group | Usual-care control with unsupervised walking advice | n=7 | 71.1±7.2 years | Men 29.9±5.2; women 24.2±2.1 kg/m² | 24 | 3 walks/week advised | Up to 30 min walking/session, excluding rests | NR | Sydney, Australia | Older adults with symptomatic PAD/intermittent claudication | ① |
|  | Experimental group | High-intensity progressive resistance training (H-PRT); low-intensity resistance training (L-RT) | H-PRT n=8; L-RT n=7 | H-PRT: 78.8±5.3; L-RT: 70.3±19.9 years | H-PRT: men 27.7±2.8, women 26.0±1.8; L-RT: men 30.6±5.7, women 21.5±3.7 kg/m² | 24 | 3 sessions/week | NR | NR | Sydney, Australia | Older adults with symptomatic PAD/intermittent claudication | ① |
| Baynard 2023 | Control group | Attention-control stretching | n=22 | 44±12 years | 29.2±7.7 kg/m² | 12 | 3 sessions/week + weekly coaching | Same contact time/duration as exercise group | 11±8 years since MS diagnosis | Chicago, Illinois, USA | Persons with multiple sclerosis; home-based aerobic training | ①；② |
|  | Experimental group | Home-based aerobic exercise (cycle ergometry) | n=26 | 49±11 years | 28.8±5.6 kg/m² | 12 | 3 sessions/week + weekly coaching | 10 min/day initially, progressed to 30 min/day | 12±8 years since MS diagnosis | Chicago, Illinois, USA | Persons with multiple sclerosis; home-based aerobic training | ①；② |
| Wang 2024 | Control group | Routine/unregulated daily exercise routine; no Tai Chi intervention | n=20 | 53.7±4.26 years | 22.0±2.23 kg/m² | 24 | NR | NR | NR | Beijing, China | Middle-aged and older postmenopausal women; Tai Chi vascular-health intervention | ②；③；④；⑤；⑥ |
|  | Experimental group | Tai Chi (BaFa WuBu) with Polar heart-rate monitoring | n=20 | 54.2±4.60 years | 23.8±3.00 kg/m² | 24 | 4 sessions/week | 60 min/session | NR | Beijing, China | Middle-aged and older postmenopausal women; Tai Chi vascular-health intervention | ②；③；④；⑤；⑥ |
| Park 2019 | Control group | No-exercise control with supervised sedentary activities | n=37 | 71.0±8.0 years | 21.3±3.2 kg/m² | 12 | Matched attendance frequency/time | 60 min/session | NR | Omaha, Nebraska, USA | Patients with peripheral artery disease; aquatic walking exercise | ①；② |
|  | Experimental group | Aquatic walking training | n=35 | 70.0±10.0 years | 20.4±2.1 kg/m² | 12 | 4 sessions/week | 60 min/session | NR | Omaha, Nebraska, USA | Patients with peripheral artery disease; aquatic walking exercise | ①；② |
| Parkington 2023 | Control group | Matched low-intensity resistance exercise without BFR | n=15 | 71.6±9.1 years | 26.5±3.5 kg/m² | 8 | 2 sessions/week | NR (5-min warm-up + matched resistance sets) | ≥0.5 years stable claudication symptoms | Sheffield, UK | Patients with PAD-related claudication; BFR feasibility trial | ① |
|  | Experimental group | Low-intensity resistance exercise with blood-flow restriction (BFR) | n=15 | 66.8±8.6 years | 27.9±3.1 kg/m² | 8 | 2 sessions/week | NR (5-min warm-up + resistance sets) | ≥0.5 years stable claudication symptoms | Sheffield, UK | Patients with PAD-related claudication; BFR feasibility trial | ① |
| Tang 2024 | Control group | Usual care + monthly online diabetes education | n=37 | 67.892±4.713 years | 24.508±3.323 kg/m² | 26 (6 months) | Monthly online education; no regular exercise | NR | 14.027±6.513 years | Haikou, Hainan, China | Elderly patients with type 2 diabetes; resistance training | ①；③；④；⑤；⑥ |
|  | Experimental group | Moderate home-based resistance training | n=37 | 65.919±3.947 years | 23.876±3.433 kg/m² | 26 (6 months) | 3 sessions/week | 40 min/session | 11.459±5.947 years | Haikou, Hainan, China | Elderly patients with type 2 diabetes; resistance training | ①；③；④；⑤；⑥ |
| Ma 2024 | Control group | Diabetes health education + follow-up only | n=31 | 56.29±5.92 years | 26.92±1.74 kg/m² | 26 (6 months) | Health education/follow-up only | 13 h education total | Categorical: 2–5/5–10/>10 years = 13/11/7 | Shaoyang, Hunan, China | Middle-aged patients with type 2 diabetes; BFR-RT vs aerobic exercise | ③；④；⑤；⑥ |
|  | Experimental group | BFR-RT; moderate-intensity aerobic exercise (AE) | BFR-RT n=32; AE n=30 | BFR-RT: 57.56±4.85; AE: 57.73±5.85 years | BFR-RT: 26.33±1.76; AE: 26.56±1.55 kg/m² | 26 (6 months) | 3 sessions/week | BFR-RT 40 min/session; AE 60 min/session | Categorical: BFR-RT 2–5/5–10/>10 years = 14/13/5; AE = 15/10/5 | Shaoyang, Hunan, China | Middle-aged patients with type 2 diabetes; BFR-RT vs aerobic exercise | ③；④；⑤；⑥ |
| Wong 2018 | Control group | No-exercise control | n=15 | 15.3 (1.1 SEM) years | 30.2 (1.2 SEM) kg/m² | 12 | No structured exercise | NR | NR | Busan, South Korea | Obese adolescent girls; combined exercise training | ② |
|  | Experimental group | Combined resistance and aerobic exercise training (CET) | n=15 | 15.2 (1.2 SEM) years | 30.1 (2.2 SEM) kg/m² | 12 | 3 sessions/week | 60 min/session | NR | Busan, South Korea | Obese adolescent girls; combined exercise training | ② |
| Mogharnasi 2017 | Control group | No-training control | n=12 | 23.7±1.76 years | 20.3±1.30 kg/m² | 8 | No training | NR | NR | Birjand, Iran | Healthy non-athlete male students; resistance vs endurance training | ③；④；⑤；⑥ |
|  | Experimental group | Resistance training (RT); endurance training (ET) | RT n=12; ET n=12 | RT: 23.7±1.13; ET: 23.5±1.07 years | RT: 20.5±1.01; ET: 20.1±0.99 kg/m² | 8 | 3 sessions/week | ET 16–30 min/session; RT session time NR | NR | Birjand, Iran | Healthy non-athlete male students; resistance vs endurance training | ③；④；⑤；⑥ |
| Martins 2010 | Control group | Sedentary control | n=13 | 81.2±7.9 years | 27.8±3.5 kg/m² | 16 | No formal exercise | NR | NR | Coimbra, Portugal | Older adults (>64 years); aerobic vs strength-based training | ①；③；④；⑤；⑥ |
|  | Experimental group | Aerobic-based training (AT); strength-based training (ST) | AT n=18; ST n=14 | AT: 76.2±7.4; ST: 73.2±6.5 years | AT: 30.1±4.7; ST: 30.8±5.3 kg/m² | 16 | 3 sessions/week | 45 min/session | NR | Coimbra, Portugal | Older adults (>64 years); aerobic vs strength-based training | ①；③；④；⑤；⑥ |

The outcome column uses coded indicators for the main outcomes extracted from each study: ① 6MWD; ② PWV; ③ TC; ④ TG; ⑤ HDL-C; ⑥ LDL-C.

# Supplementary Table S4 Details of interventions conducted in the trial.

| **Study** | **Group 1** | **Group 2** | **Group 3** |
| --- | --- | --- | --- |
| Oliveira 2015 | Participants in the experimental group received an exercise-based cardiac rehabilitation program for 8 weeks. The supervised sessions were performed 3 times a week, consisting of 10 min of warm-up, 30 min of aerobic exercise on a cycloergometer or treadmill at 70-85% of maximal heart rate, and 10 min of cool-down. Participants also received usual medical care and general nutritional counseling. | Participants in the control group received only usual medical care, which included regular appointments with a cardiologist and optimized medication. They also received general nutritional counseling at the beginning and the end of the program period. | NA. |
| Manfredini 2024 | The participants in the experimental group received the TiTo-SHB structured home-based pain-free interval walking therapy. The exercise sessions were conducted 2 times a day, 6 days a week, for a total duration of 24 weeks. Each session lasted for a total of 16 min, which included 8 min of actual walking interspersed with sitting. Walking speed was maintained at home using a metronome and progressively increased by approximately 3 steps/min every 2 weeks. | Participants in the control group received traditional "go home and walk" walking advice. They were instructed to perform walking exercises to achieve 30 min of walking at least 3 days a week over the 24-week intervention period. When facing claudication pain, they were allowed to rest and resume walking as soon as possible. | NA. |
| Kearney 2014 | The participants in the walking group were required to incorporate three 10-minute bouts of brisk walking into their daily routine on 5 days per week for 6 months. Brisk walking was described as a pace that left them slightly out of breath but still able to maintain a conversation. Participants were also contacted by telephone either monthly or weekly to provide support and encouragement. | Participants in the control group were given light stretching exercises to complete twice daily on 5 days per week for 6 months. They were contacted on a monthly basis to control for the effects of telephone contact. | NA. |
| Son 2025 | The exercise group participated in a supervised circuit training program 3 times per week for 12 weeks. The sessions included 10 min of static stretching for warm-up, a main exercise circuit (push-ups, squats, lunges, kickbacks, shoulder presses, pull-ups, hip-bridges, leg-raises, and crunches), and 10 min of static stretching for cool-down. The main exercise lasted 35 min at 60-70% of heart rate reserve during weeks 1-6, and increased to 45 min at 70-80% of heart rate reserve during weeks 7-12. Each exercise was completed within 60 s with 50 s of rest between sets. | Participants in the control group did not engage in any structured physical activity. They were asked to maintain their usual lifestyle throughout the 12-week study period. | NA. |
| Bronas 2011 | The arm-ergometry group received supervised arm-ergometer exercise training 3 times weekly for 12 weeks. Each session lasted 60 minutes, which included 5 minutes of warm-up and cool-down low-intensity exercises. The exercise was performed intermittently, beginning with 2-minute increments of exercise followed by 2-minute rest periods, and progressively increased to up to 5 minutes of continuous exercise and 1 minute of rest. | The treadmill-walking group received supervised treadmill-walking training 3 times weekly for 12 weeks. Each session lasted 60 minutes, including 5 minutes of warm-up and cool-down. Participants intermittently walked until they experienced moderately severe claudication, stopped to rest while seated until the pain subsided, and then resumed walking. | Participants in the control group were instructed to continue to follow the care prescribed by their primary physicians and received written instructions on how to exercise independently. They visited the exercise laboratory once each week for 12 weeks to monitor resting heart rate and blood pressure. |
| Lin 2021 | The experimental group engaged in a general rehabilitation course combined with supervised hospital-based cycling exercise training on a bicycle ergometer. The cycling sessions were performed 3 days a week for 12 weeks. The protocol comprised a warm-up at 30% of peak oxygen consumption for 3 minutes, continuous cycling at the ventilation threshold for 30 minutes, and a cool-down at 30% of peak oxygen consumption for 3 minutes. | Participants in the control group only engaged in the general peripheral arterial disease rehabilitation course for 12 weeks. They followed the instructions provided by their rehabilitation physicians without receiving the cycling exercise training. | NA. |
| McDermott 2019 | The supervised treadmill exercise group performed treadmill exercise 3 times weekly for 6 months under the supervision of an exercise physiologist. Sessions began with 15 minutes and increased to 40 minutes of exercise. Participants were encouraged to exercise to near-maximal leg symptoms. Between months 6 and 12, they received telephone calls to encourage continued walking exercise at home or other venues. | The supervised resistance training group exercised 3 times weekly for 6 months with a trainer. They performed 3 sets of 8 repetitions of knee-extension, leg-press, leg-curl, squat, and toe-rise exercises. Exercise intensity increased from 50% to 80% of their 1-repetition maximum. Between months 6 and 12, they were provided with ankle weights and instructed to perform exercises at home. | The attention control group attended 11 1-hour group sessions over a 6-month period, receiving information from a registered dietician regarding nutritional supplements, healthy restaurant eating, and increasing fruit and vegetable consumption. During months 6 to 12, they were telephoned by the dietician to review the nutrition information. |
| McDermott 2025 | The supervised treadmill exercise group received treadmill exercise 3 times weekly with an exercise physiologist for 6 months. Walking exercise duration was increased gradually until 50 minutes of exercise per session was achieved. Participants were asked to walk to induce maximal symptoms of leg tightness or cramping, stop to rest, and resume walking after symptoms subsided. | Participants in the health education control group attended weekly 1-hour educational sessions for 6 months. The sessions covered health topics of interest to the typical patient with peripheral artery disease, including cancer screening, immunizations, and hypertension. | NA. |
| Jia 2025 | The first group participated in High-Intensity Resistance Training (HIRT) 3 times a week for 12 weeks. After a 5-minute treadmill walking warm-up, participants performed resistance exercises including high pull-downs, shoulder presses, upward inclined barbell bench presses, leg raises, seated leg curls, and seated leg bends and extensions. The intensity was set at 70% of their 1-repetition maximum, with each session lasting approximately 70 min. | The second group participated in High-Intensity Resistance Training combined with Aerobic Exercise Training (HIR-AET) 3 times a week for 12 weeks. Following a 5-minute warm-up, participants completed 4 sets of 10 repetitions of the resistance exercises at 70% of their 1-repetition maximum for about 60 min. This was followed by 15 min of jogging at an intensity of 60% of their peak oxygen uptake. | The third group participated in Low-Intensity Resistance Training combined with Blood Flow Restriction Training (LIR-BFRT) 3 times a week for 12 weeks. After a 5-minute warm-up, a compression band was strapped around the upper arms and thighs. Participants performed 4 sets of 10 repetitions of resistance exercises at 30% of their 1-repetition maximum, with 3-minute intervals for loosening the pressurized band after each set. |
| Bearne 2022 | The experimental group received a home-based, walking exercise behavior change intervention delivered by trained physical therapists. Over 3 months, participants attended two 60-minute in-person sessions and two 20-minute telephone sessions. They collaboratively set goals to achieve at least 30 minutes of walking per day, at a pace eliciting moderate leg symptoms, 3 times per week. Participants received a pedometer, an intervention manual, and an exercise diary to self-monitor their behavior. | Participants randomized to the usual care group received no study intervention. They solely received the standard care provided by their vascular specialists. | NA. |
| Baynard 2023 | Participants in the aerobic exercise group participated in a 12-week home-based cycle ergometry intervention for 3 days a week. Exercise started at 10 min/day and increased by 5 min/day weekly until achieving 30 min/day. The intensity began at 40% of peak aerobic capacity and increased by 5% weekly until reaching 60-65%. Participants received weekly behavioral coaching via phone or video chats and were instructed to not engage in structured exercise outside the study. | Participants in the attention-control group performed a standardized stretching protocol at home 3 days a week for 12 weeks. They received the same contact time and weekly behavioral coaching via phone or video chats. They were instructed not to undertake any structured activities outside of the study. | NA. |
| McDermott 2021 | Participants in the low-intensity exercise group performed home-based walking 5 times per week for up to 50 minutes per session for 12 months. They walked at a comfortable pace without inducing ischemic leg symptoms. They wore an accelerometer to monitor their exercise intensity and uploaded the data to a study website. A coach telephoned them weekly to review the data and help them adhere to their prescribed exercise. | Participants in the high-intensity exercise group performed home-based walking 5 times per week for up to 50 minutes per session for 12 months. They walked at a pace eliciting moderate to severe ischemic leg symptoms. They also wore an accelerometer to monitor intensity and received weekly telephone calls from a coach to review data and support adherence. | Participants in the nonexercise control group attended weekly 1-hour educational sessions at the medical center during the first 4 weeks. During weeks 5 through 52, they received weekly educational telephone calls discussing health topics. |
| Ohta 2012 | Participants in the bench step exercise group practiced a home-based bench step exercise program for 12 weeks. They exercised for 10 to 20 minutes, 3 times daily, amounting to a total of 140 minutes per week. The step height was between 15 cm and 20 cm, and the intensity was set at the individual's lactate threshold. Participants recorded their durations in training logs and attended weekly sessions at a community center to promote compliance. | Participants in the control group were asked to maintain an ordinary lifestyle during the 12-week study period. They were invited to take part in the bench step exercise program after the study concluded. | NA. |
| Woo 2004 | Participants in the diet plus exercise group received a balanced hypocaloric diet of 900 to 1200 kcal daily and participated in a supervised structured exercise program. The exercise was twice per week for the first 6 weeks, then once weekly for 1 year. Each 75-minute session included 10 minutes of warm-up, 30 minutes of resistance training, 10 minutes of aerobic exercise at 60% to 70% of maximum heart rate, 10 minutes of agility training, and 5 minutes of cool-down. | Participants in the diet only group received a balanced hypocaloric diet providing 900 to 1200 kcal daily, which was low in fat and high in complex carbohydrates. They were interviewed by a dietitian twice weekly for the first 6 weeks, and bimonthly subsequently for 1 year. They were instructed to maintain their usual lifestyle activities. | NA. |
| Ratajczak 2019 | Participants in the endurance training group completed 3 training sessions per week for 3 months on cycle ergometers. Each session consisted of a 5-minute warm-up with stretching exercises at 50-60% of maximum heart rate, 45 minutes of endurance training at 60-80% of maximum heart rate, 5 minutes of non-weight-bearing cycling, and a 5-minute cool-down with stretching and breathing exercises. | Participants in the combined training group completed an endurance-strength program 3 times per week for 3 months. Sessions included a 5-minute warm-up, 20 minutes of strength exercises using a neck barbell and gymnastics ball, 25 minutes of endurance exercise on cycle ergometers at 60-80% of maximum heart rate, 5 minutes of non-weight-bearing cycling, and a 5-minute cool-down. | NA. |
| Park 2020 | Participants in the heated-water exercise therapy group participated in supervised sessions 4 days a week for 60 minutes a day over 12 weeks. The sessions were performed in water heated to 30-31°C and included a 10-minute warm-up, 40 minutes of main exercise, and a 10-minute cool-down. Exercise intensity progressively increased from 50-60% to 70-85% of their heart rate reserve over the 12 weeks. | Participants in the land-based exercise therapy group performed a supervised treadmill walking program 4 days a week for 60 minutes a day over 12 weeks. Sessions consisted of a 10-minute warm-up, 40 minutes of main exercise involving stepping movements and treadmill walking, and a 10-minute cool-down. The intensity was matched to the water group, progressing from 50-60% to 70-85% of heart rate reserve. | NA. |
| Correia 2020 | Participants in the isometric handgrip training group performed 3 home-based sessions per week for 8 weeks. The protocol consisted of 4 sets of 2-minute unilateral isometric handgrip holds at 30% of maximum voluntary contraction with a 4-minute rest interval between repetitions. They used a device providing visual and audible feedback and received biweekly phone calls for reinforcement. They were also instructed to increase their general daily physical activity to meet standard recommendations. | Participants in the control group received a compression ball to perform sham training, minimizing placebo effects. They were recommended to perform 3 sessions per week consisting of 3 sets of 10 compressions per hand with a 1-minute rest interval between sets. Like the intervention group, they were instructed to increase general daily physical activity to meet standard recommendations. | NA. |
| Laslovich 2020 | Participants in the physical activity sedentary reduction group participated in an interactive 12-week online program to increase lifestyle physical activity and reduce sedentary behavior. They wore an activity tracker that provided real-time visual feedback and vibration alerts after 50 minutes of continuous inactivity. They were instructed to incorporate at least two 10-minute bouts of continuous self-paced walking daily and watched six bimonthly online health videos. | Participants in the attention control group received the same six bimonthly online videos containing health recommendations related to peripheral arterial disease. They were asked to simply continue their normal daily activities and routines during the 12-week study period. | NA. |
| Gardner 2023 | Participants in the home exercise program group performed 3 months of intermittent walking to mild-to-moderate claudication pain at least 3 days per week at a self-selected pace. They monitored their exercise using a step activity monitor. Afterward, they continued this home program for an additional 15 months, meeting with research staff every 3 months to download data and discuss strategies to overcome exercise barriers. | Participants in the supervised exercise group completed 3 months of intermittent treadmill walking 3 days per week, with sessions progressing from 15 to 40 minutes. After the initial 3 months, they transitioned to the home exercise program for an additional 15 months, meeting with research staff every 3 months for feedback and data review. | Participants in the control group performed supervised attention-control light resistance training 3 times per week for the first 3 months without any walking exercise. After the initial 3 months, they were given walking advice without further supervision and transitioned to usual care for the remaining 15 months. |
| Parmenter 2013 | Participants in the high-intensity progressive resistance training group completed 24 weeks of supervised whole-body weight lifting 3 times per week. They performed 3 sets of 8 repetitions for seven major muscle groups. Intensity was set at 50% of their 1-repetition maximum and increased to 80%, with regular adjustments. They also received written standard lifestyle advice. | Participants in the low-intensity resistance training group completed 24 weeks of supervised training 3 times per week. They performed the same exercises, but the intensity started at 20% of their 1-repetition maximum and increased until reaching 30% of their baseline maximum, where it was maintained for the rest of the trial. They also received standard lifestyle advice. | Participants in the usual care control group received written standard lifestyle advice and were instructed to walk outside unsupervised 3 times per week. They were asked to walk at their usual speed to the maximum tolerable pain level, rest, and repeat for up to 30 minutes, recording their walks in a diary. |
| Zeng 2024 | Participants in the experimental group practiced health Qigong combined with Tibetan dance for 12 weeks, 3 times per week, with each session lasting 60 minutes. The session included a 10-minute warm-up, 40 minutes of basic movements supervised by a researcher to music, and a 10-minute relaxation/stretching phase. | Participants in the control group received no exercise intervention and were instructed to continue their regular routines. | NA. |
| Wang 2024 | Participants in the exercise group underwent a 24-week Tai Chi (BaFa WuBu) training intervention, 4 sessions per week, for 60 minutes per session. The training was divided into basic, improvement, consolidation, and proficiency phases. Real-time heart rate monitoring using a Polar H10 chest strap was used to maintain the heart rate at approximately 60% of the maximum heart rate. | Participants in the control group underwent a routine of an unregulated exercise workout as per their daily routine without Tai Chi intervention. | NA. |
| Park 2019 | Participants in the aquatic walking training group (AQ) performed supervised sessions in waist-to-chest-deep water (28-30°C) for 12 weeks, 4 days per week, for 60 minutes per day. The session included a 10-minute warm-up, 40 minutes of main exercises (lower limb movements and water walking), and a 10-minute cool-down. Intensity progressed from 50-60% to 70-85% of heart rate reserve. | Participants in the control group (CON) did not participate in any exercise program. They were supervised in the laboratory at the same frequency and time of day performing sedentary activities such as reading books or listening to music. | NA. |
| Parkington 2023 | Participants in the blood flow restriction (BFR) group received supervised resistance exercise twice weekly for 8 weeks. They performed 4 sets of leg press and 3 sets of knee extension at 20% 1RM. A pneumatic cuff was inflated around the proximal aspect of the legs at 50% arterial occlusion pressure 10 seconds before exercise, remained inflated during rest periods, and deflated immediately after. | Participants in the control group received a matched supervised exercise program (same leg press and knee extension exercises, volume, and load) but without the application of blood flow restriction. | NA. |
| Tang 2024 | Participants in the resistance training group performed home-based resistance exercise 3 times a week for 6 months using an elastic band. Each 40-minute session included a 5-minute warm-up, 30 minutes of resistance exercises (progressing from 45% to 55% of 1RM), and a 5-minute cool-down. They also attended monthly online group educational sessions. | Participants in the control group did not engage in regular exercise but attended monthly online group educational sessions on diabetes self-management. They were instructed to record their daily physical activities using WeChat. | NA. |
| Ma 2024 | Participants in the aerobic exercise (AE) group received supervised training 3 times per week for 6 months. Each 60-minute session included a 5-10 minute warm-up, 45-50 minutes of moderate-intensity AE (40-59% of heart rate reserve), and 5 minutes of stretching. | Participants in the blood flow-restrictive resistance training (BFR-RT) group trained 3 times per week for 6 months, 40 minutes per session. They used KAATSU Air Bands at 40-50% limb occlusion pressure to perform 4 sets of resistance training at 20-40% 1RM with a total restriction time of 6.5 minutes per cycle. | Participants in the control group received health education and follow-up but were not provided with a detailed exercise program or supervised exercise interventions. |
| Wong 2018 | Participants in the combined exercise training (CET) group trained for 60 minutes per day, 3 times a week for 12 weeks. The session consisted of 5 minutes of warm-up, 20 minutes of resistance band exercises, 30 minutes of treadmill walking, and a 5-minute cool-down. The intensity gradually increased from 40-50% to 60-70% of heart rate reserve. | Participants in the control group were instructed to refrain from any mode of structured exercise training during the 12-week study period. | NA. |
| Mogharnasi 2017 | Participants in the endurance training (ET) group performed treadmill-running 3 days/week for 8 weeks. The duration increased from 16 minutes in the first week to 30 minutes by the eighth week, and intensity increased from 65% to 80% of maximum heart rate. | Participants in the resistance training (RT) group exercised 3 days/week for 8 weeks. The program consisted of 3 sets of 8-10 repetitions at 60-80% of 1RM, with 2-minute rests, covering large muscles of the upper and lower body. | Participants in the control group did not perform the structured training program and maintained their ordinary lifestyle. |
| Martins 2010 | Participants in the aerobic-based training (AT) group exercised 3 times per week for 16 weeks. Sessions were 45 minutes long, starting at 40-50% of heart rate reserve and increasing progressively up to 71-85% of heart rate reserve. | Participants in the strength-based training (ST) group completed 45-minute sessions 3 times per week for 16 weeks. The program used callisthenic exercises and elastic bands for 8 major muscle groups, progressing from 1 set of 8 repetitions to 3 sets of 15 repetitions. | Participants in the control group remained sedentary throughout the study (without formal exercise) but maintained their lifestyle routines. |

**Supplementary Table S5: Exercise Therapy Classification.**

| **NO.** | **Types of Exercise Interventions** | **Abbreviation** | **Definition of Exercise Intervention** |
| --- | --- | --- | --- |
| 1 | Conventional Treatment | CT | Standard medical rehabilitation interventions including pharmacological treatments, physiotherapy, and occupational therapy based on evidence-based clinical practice guidelines. |
| 2 | Aerobic Exercise | AE | A form of continuous, rhythmic physical activity that elevates heart rate and utilizes large muscle groups. Examples include cycling, jogging, swimming, and walking. This exercise can be sustained without causing exhaustion and provides sufficient oxygen to muscles through the cardiopulmonary oxygen supply system. |
| 3 | Resistance Training | RT | Muscle contraction against external resistance, aimed at enhancing muscle strength, endurance, or athletic performance. Common examples include weightlifting, push-ups, and pull-ups. |
| 4 | Isometric Handgrip Exercise | IHE | Muscle contraction without movement of the affected body part. Participants perform multiple, timed, constant handgrip contractions at a set percentage (typically 30%) of maximum voluntary contraction (MVC), with timed rest intervals between contractions. |
| 5 | Aerobic Exercise + Resistance Training | AERT | A combined training model integrating aerobic and resistance exercises to simultaneously improve cardiovascular endurance and muscular strength/endurance. |
| 6 | Qigong + Dancing | QD | Qigong is an ancient Chinese health practice that enhances physical and mental well-being through breath regulation, posture, mental focus, and body massage. Combined with dance movements, it forms a mind-body rehabilitation exercise suitable for patients unable to perform high-intensity physical activities. |
| 7 | Supervision + Aerobic Exercise | SAE | Aerobic exercise conducted under the guidance and monitoring of professionals (e.g., physiotherapists, exercise instructors) to improve adherence, safety, and exercise prescription effectiveness. |
| 8 | Supervision + Resistance Exercise | SRE | Resistance training conducted under professional supervision to ensure proper technique, appropriate intensity, reduced injury risk, and improved training outcomes. |
| 9 | Tai Chi Chuan | TCC | A traditional Chinese exercise form featuring slow, flowing movements emphasizing breath regulation, body balance, and internal energy cultivation. Tai Chi is used in rehabilitation to improve balance function, gait speed, mood status, and cognitive function. |
| 10 | Resistance Exercise + Blood Flow Restriction | BFRT | A specialized training method where proximal limb compression devices (e.g., inflatable cuffs) restrict venous return without affecting arterial perfusion, while performing low-load resistance exercise. This approach provides sufficient metabolic stimulation for muscle growth and strength enhancement while reducing joint stress. |
| 11 | Heat Treat Aerobic Exercise | HTAE | Thermotherapy is a physical treatment that increases tissue metabolism, blood flow, inflammation response, and connective tissue extensibility. Combined with aerobic exercise, it synergistically improves pain, muscle spasm, and joint function, commonly used in musculoskeletal injury and osteoarthritis rehabilitation. |
| 12 | Stretching Exercise | SE | A slow, sustained muscle relaxation process aimed at restoring joint range of motion and relieving muscle tension. Methods include static stretching (10-30 seconds), dynamic stretching, and proprioceptive neuromuscular facilitation (PNF). Stretching can be active or passive, widely applied in rehabilitation and athletic performance enhancement. |

**References**

[1] Makita, S., Yasu, T., Akashi, Y. J., Adachi, H., Izawa, H., Ishihara, S., et al. (2022). JCS/JACR 2021 Guideline on Rehabilitation in Patients With Cardiovascular Disease. Circ J 87, 155–235. doi: 10.1253/circj.CJ-22-0234

[2] Tessler, J., Ahmed, I., and Bordoni, B. (2025). “Cardiac Rehabilitation,” in StatPearls. Available at: https://www.ncbi.nlm.nih.gov/books/NBK537196/.

[3] World Health Organization (2020). “GLOSSARY OF TERMS,” in WHO Guidelines on Physical Activity and Sedentary Behaviour. Available at: https://www.ncbi.nlm.nih.gov/books/NBK566047/.

[4] Esco, M. R. (2013). Resistance Training for Health and Fitness. American College of Sports Medicine.

[5] Ogbutor, G. U., Nwangwa, E. K., and Uyagu, D. D. (2019). Isometric handgrip exercise training attenuates blood pressure in prehypertensive subjects at 30% maximum voluntary contraction. Niger J Clin Pract 22, 1765–1771. doi: 10.4103/njcp.njcp_240_18

[6] Jahnke, R., Larkey, L., Rogers, C., Etnier, J., and Lin, F. (2010). A comprehensive review of health benefits of qigong and tai chi. Am J Health Promot 24, e1–e25. doi: 10.4278/ajhp.081013-LIT-248

[7] Gerhard-Herman, M. D., Gornik, H. L., Barrett, C., Barshes, N. R., Corriere, M. A., Drachman, D. E., et al. (2017). 2016 AHA/ACC Guideline on the Management of Patients With Lower Extremity Peripheral Artery Disease: A Report of the American College of Cardiology/American Heart Association Task Force on Clinical Practice Guidelines. Circulation 135. doi: 10.1161/CIR.0000000000000471

[8] National Center for Complementary and Integrative Health (2023). Tai Chi: What You Need To Know. NCCIH. Available at: https://www.nccih.nih.gov/health/tai-chi-what-you-need-to-know.

[9] Lorenz, D. S., Bailey, L., Wilk, K. E., Mangine, R. E., Head, P., Grindstaff, T. L., et al. (2021). Blood Flow Restriction Training. Journal of Athletic Training 56, 937–944. doi: 10.4085/418-20

[10] Cognetti, D. J., Sheean, A. J., and Owens, J. G. (2022). Blood Flow Restriction Therapy and Its Use for Rehabilitation and Return to Sport: Physiology, Application, and Guidelines for Implementation. Arthroscopy, Sports Medicine, and Rehabil. 4. doi: 10.1016/j.asmr.2021.09.025

[11] Mason, P. (2014). Thermotherapy and cryotherapy. The Pharmaceutical Journal. Available at: https://pharmaceutical-journal.com/article/ld/thermotherapy-and-cryotherapy.

[12] Page, P. (2012). Current concepts in muscle stretching for exercise and rehabilitation. Int J Sports Phys Ther 7, 109–119.

# Supplementary Table S6 Results of pairwise comparisons between groups for 6MWD.

| RT |  |  |  |  |
| --- | --- | --- | --- | --- |
| 12.12 (-28.61,52.85) | AE |  |  |  |
| 18.68 (-104.55,141.91) | 6.56 (-113.15,126.27) | SRE |  |  |
| 32.83 (-17.64,83.29) | 20.70 (-31.78,73.19) | 14.14 (-113.42,141.71) | SAE |  |
| 56.42 (15.94,96.91) | 44.30 (16.30,72.30) | 37.74 (-78.65,154.13) | 23.60 (-28.62,75.82) | CT |

Results from the NMA, including mixed network comparisons and indirect comparisons, are presented in the lower left triangle. The data are presented as the mean difference with 95% confidence interval, MD (95% CI). An MD less than 0 indicates that the intervention may reduce the outcome value compared with the comparator. A deeper background color indicates statistical significance. RT: Resistance Training; AE: Aerobic Exercise; SRE: Supervised Resistance Exercise; SAE: Supervised Aerobic Exercise; CT: Conventional Therapy.

**Supplementary Table S7: The SUCRA Values of 6MWD**

| Treatm~t | SUCRA | PrBest | MeanRank |
| --- | --- | --- | --- |
| CT | 11.4 | 0 | 4.5 |
| AE | 65.2 | 15.7 | 2.4 |
| RT | 80.6 | 44.1 | 1.8 |
| SAE | 38.1 | 4.4 | 3.5 |
| SRE | 54.7 | 35.8 | 2.8 |

Note:CT：Conventional Treatment；AE：Aerobic Exercise；RT：Resistance Training；SAE：Supervision + Aerobic Exercise ；SRE：Supervision + Resistance Exercise

# Supplementary Table S8: Results of pairwise comparisons between groups for PWV.

| AE |  |  |  |  |  |  |
| --- | --- | --- | --- | --- | --- | --- |
| 0.01 (-3.73,3.75) | QD |  |  |  |  |  |
| -0.66 (-2.34,1.02) | -0.67 (-4.42,3.08) | AERT |  |  |  |  |
| -1.52 (-2.70,-0.34) | -1.53 (-5.08,2.02) | -0.86 (-2.06,0.33) | CT |  |  |  |
| -2.12 (-4.53,0.29) | -2.13 (-6.26,2.00) | -1.46 (-3.88,0.96) | -0.60 (-2.70,1.50) | IHE |  |  |
| -2.98 (-6.65,0.69) | -2.99 (-8.23,2.25) | -2.32 (-6.35,1.71) | -1.46 (-5.31,2.39) | -0.86 (-5.25,3.53) | SE |  |
| -5.31 (-7.46,-3.17) | -5.32 (-9.30,-1.34) | -4.65 (-6.81,-2.50) | -3.79 (-5.59,-2.00) | -3.19 (-5.96,-0.43) | -2.33 (-6.58,1.92) | TCC |

Results from the NMA, including mixed network comparisons and indirect comparisons, are presented in the lower left triangle. The data are presented as the mean difference with 95% confidence interval, MD (95% CI). An MD less than 0 indicates that the intervention may reduce the outcome value compared with the comparator. A deeper background color indicates statistical significance. AE: Aerobic Exercise; QD: Qigong and Dance Exercise; AERT: Aerobic Exercise combined with Resistance Training; CT: Conventional Therapy; IHE: Isometric Handgrip Exercise; SE: Stretching Exercise; TCC: Tai Chi Chuan

**Supplementary Table S9: The SUCRA Values of PWV.**

| Treatm~t | SUCRA | PrBest | MeanRank |
| --- | --- | --- | --- |
| CT | 45.9 | 0 | 4.2 |
| AE | 85.9 | 37.9 | 1.8 |
| IHE | 38 | 1.8 | 4.7 |
| AERT | 70.7 | 9 | 2.8 |
| QD | 77.9 | 47.8 | 2.3 |
| TCC | 2.6 | 0 | 6.8 |
| SE | 29 | 3.5 | 5.3 |

Note：CT：Conventional Treatment；AE：Aerobic Exercise；IHE：Isometric Handgrip Exercise；AERT：Aerobic Exercise + Resistance Training；QD：Qigong + Dancing；TCC: Tai Chi Chuan；SE：Stretching Exercise

# Supplementary Table S10: Results of pairwise comparisons between groups for TC.

| QD |  |  |  |  |  |  |  |
| --- | --- | --- | --- | --- | --- | --- | --- |
| -1.24 (-1.95,-0.53) | BFRT |  |  |  |  |  |  |
| -1.31 (-2.00,-0.62) | -0.06 (-0.61,0.49) | RT |  |  |  |  |  |
| -1.36 (-2.13,-0.59) | -0.12 (-0.69,0.46) | -0.06 (-0.68,0.57) | SE |  |  |  |  |
| -1.41 (-2.04,-0.79) | -0.17 (-0.52,0.19) | -0.11 (-0.54,0.32) | -0.05 (-0.50,0.40) | AE |  |  |  |
| -1.46 (-1.99,-0.93) | -0.22 (-0.68,0.25) | -0.15 (-0.59,0.28) | -0.10 (-0.65,0.46) | -0.05 (-0.37,0.28) | CT |  |  |
| -1.53 (-2.16,-0.89) | -0.28 (-0.82,0.26) | -0.22 (-0.75,0.31) | -0.16 (-0.78,0.45) | -0.11 (-0.53,0.30) | -0.07 (-0.42,0.28) | AERT |  |
| -2.58 (-3.45,-1.71) | -1.34 (-2.17,-0.50) | -1.27 (-2.09,-0.46) | -1.22 (-2.10,-0.33) | -1.17 (-1.93,-0.41) | -1.12 (-1.81,-0.43) | -1.05 (-1.82,-0.28) | TCC |

Results from the NMA, including mixed network comparisons and indirect comparisons, are presented in the lower left triangle. The data are presented as the mean difference with 95% confidence interval, MD (95% CI). An MD less than 0 indicates that the intervention may reduce the outcome value compared with the comparator. A deeper background color indicates statistical significance. QD: Qigong and Dance Exercise; BFRT: Blood Flow Restriction Training; RT: Resistance Training; SE: Stretching Exercise; AE: Aerobic Exercise; CT: Conventional Therapy; AERT: Aerobic Exercise combined with Resistance Training; TCC: Tai Chi Chuan

**Supplementary Table S11: The SUCRA Values of TC**

| Treatm~t | SUCRA | PrBest | MeanRank |
| --- | --- | --- | --- |
| CT | 40.1 | 0 | 5.2 |
| AE | 45.7 | 0 | 4.8 |
| RT | 60.6 | 0 | 3.8 |
| AERT | 33.4 | 0 | 5.7 |
| QD | 100 | 100 | 1 |
| TCC | 0.1 | 0 | 8 |
| BFRT | 67.9 | 0 | 3.2 |
| SE | 52.3 | 0 | 4.3 |

Note：CT：Conventional Treatment；AE：Aerobic Exercise；RT：Resistance Training；AERT：Aerobic Exercise + Resistance Training；QD：Qigong + Dancing；TCC: Tai Chi Chuan；BFRT：Resistance Exercise + Blood Flow Restriction；HTAE：Heat Treat Aerobic Exercise；SE：Stretching Exercise

# Supplementary Table S12: Results of pairwise comparisons between groups for TG.

| QD |  |  |  |  |  |
| --- | --- | --- | --- | --- | --- |
| -0.59 (-1.77,0.60) | AE |  |  |  |  |
| -0.58 (-1.90,0.74) | 0.01 (-0.62,0.64) | BFRT |  |  |  |
| -0.80 (-2.12,0.52) | -0.21 (-0.89,0.46) | -0.22 (-1.10,0.66) | RT |  |  |
| -0.84 (-2.00,0.32) | -0.25 (-0.51,0.00) | -0.26 (-0.88,0.36) | -0.04 (-0.67,0.58) | CT |  |
| -0.96 (-2.21,0.29) | -0.37 (-0.90,0.16) | -0.38 (-1.16,0.40) | -0.16 (-0.94,0.62) | -0.12 (-0.58,0.34) | TCC |

Results from the NMA, including mixed network comparisons and indirect comparisons, are presented in the lower left triangle. The data are presented as the mean difference with 95% confidence interval, MD (95% CI). An MD less than 0 indicates that the intervention may reduce the outcome value compared with the comparator. A deeper background color indicates statistical significance. QD: Qigong and Dance Exercise; AE: Aerobic Exercise; BFRT: Blood Flow Restriction Training; RT: Resistance Training; CT: Conventional Therapy; TCC: Tai Chi Chuan.

**Supplementary Table S13: The SUCRA Values of TG.**

| Treatm~t | SUCRA | PrBest | MeanRank |
| --- | --- | --- | --- |
| CT | 29.6 | 0 | 4.5 |
| AE | 65.3 | 6.5 | 2.7 |
| RT | 37.8 | 4.8 | 4.1 |
| QD | 87.6 | 74.3 | 1.6 |
| TCC | 19.6 | 0.8 | 5 |
| BFRT | 60 | 13.6 | 3 |

Note：CT：Conventional Treatment；AE：Aerobic Exercise；RT：Resistance Training；QD：Qigong + Dancing；TCC: Tai Chi Chuan；BFRT：Resistance Exercise + Blood Flow Restriction

# Supplementary Table S14: Results of pairwise comparisons between groups for HDL-C.

| QD |  |  |  |  |  |
| --- | --- | --- | --- | --- | --- |
| 0.51 (0.24,0.77) | RT |  |  |  |  |
| 0.61 (0.38,0.84) | 0.10 (-0.04,0.24) | CT |  |  |  |
| 0.73 (0.49,0.98) | 0.22 (0.08,0.37) | 0.12 (0.03,0.22) | AE |  |  |
| 0.79 (0.50,1.07) | 0.28 (0.06,0.50) | 0.18 (0.00,0.35) | 0.06 (-0.13,0.24) | AERT |  |
| 0.82 (0.44,1.20) | 0.31 (-0.03,0.65) | 0.21 (-0.10,0.52) | 0.09 (-0.24,0.41) | 0.03 (-0.32,0.39) | TCC |

Results from the NMA, including mixed network comparisons and indirect comparisons, are presented in the lower left triangle. The data are presented as the mean difference with 95% confidence interval, MD (95% CI). An MD greater than 0 indicates that the intervention may increase the outcome value compared with the comparator. A deeper background color indicates statistical significance. QD: Qigong and Dance Exercise; RT: Resistance Training; CT: Conventional Therapy; AE: Aerobic Exercise; AERT: Aerobic Exercise combined with Resistance Training; TCC: Tai Chi Chuan

**Supplementary Table S15: The SUCRA Values of HDL-C.**

| Treatm~t | SUCRA | PrBest | MeanRank |
| --- | --- | --- | --- |
| CT | 59.2 | 0 | 3 |
| AE | 28.8 | 0 | 4.6 |
| RT | 77.5 | 0 | 2.1 |
| AERT | 17.5 | 0 | 5.1 |
| QD | 100 | 100 | 1 |
| TCC | 16.9 | 0 | 5.2 |

Note：CT：Conventional Treatment；AE：Aerobic Exercise；RT：Resistance Training；AERT：Aerobic Exercise + Resistance Training；QD：Qigong + Dancing；TCC: Tai Chi Chuan

# Supplementary Table S16: Results of pairwise comparisons between groups for LDL-C.

| BFRT |  |  |  |  |  |
| --- | --- | --- | --- | --- | --- |
| -0.25 (-1.16,0.65) | AE |  |  |  |  |
| -0.31 (-1.21,0.60) | -0.05 (-0.51,0.40) | CT |  |  |  |
| -0.36 (-1.61,0.90) | -0.10 (-1.03,0.83) | -0.05 (-0.98,0.88) | AERT |  |  |
| -0.63 (-1.72,0.47) | -0.37 (-1.06,0.32) | -0.32 (-1.00,0.37) | -0.27 (-1.38,0.84) | RT |  |
| -0.78 (-2.31,0.75) | -0.52 (-1.84,0.79) | -0.47 (-1.70,0.76) | -0.42 (-1.96,1.12) | -0.15 (-1.56,1.26) | TCC |

Results from the NMA, including mixed network comparisons and indirect comparisons, are presented in the lower left triangle. The data are presented as the mean difference with 95% confidence interval, MD (95% CI). An MD less than 0 indicates that the intervention may reduce the outcome value compared with the comparator. A deeper background color indicates statistical significance. BFRT: Blood Flow Restriction Training; AE: Aerobic Exercise; CT: Conventional Therapy; AERT: Aerobic Exercise combined with Resistance Training; RT: Resistance Training; TCC: Tai Chi Chuan

**Supplementary Table S17: The SUCRA Values of LDL-C.**

| Treatm~t | SUCRA | PrBest | MeanRank |
| --- | --- | --- | --- |
| CT | 56.3 | 6.1 | 3.2 |
| AE | 62 | 10 | 2.9 |
| RT | 27.8 | 3.4 | 4.6 |
| AERT | 50.9 | 19 | 3.5 |
| TCC | 25.5 | 8.7 | 4.7 |
| BFRT | 77.6 | 52.8 | 2.1 |

Note：CT：Conventional Treatment；AE：Aerobic Exercise；RT：Resistance Training；AERT：Aerobic Exercise + Resistance Training；TCC: Tai Chi Chuan；BFRT：Resistance Exercise + Blood Flow Restriction

**Supplementary Figure S1: Risk of bias assessment included in the study.**

**Supplementary Figure S2: Results of pairwise comparisons between groups for 6MWD.**

Note:CT：Conventional Treatment；AE：Aerobic Exercise；RT：Resistance Training；SAE：Supervision + Aerobic Exercise ；SRE：Supervision + Resistance Exercise

**Supplementary Figure S3:** **Pooled Forest Plot for 6MWD scores, AE and CT, RT and CT, SAE and CT, as well as SRE and CT.**

Note:AE：Aerobic Exercise；RT：Resistance Training；SAE：Supervision + Aerobic Exercise ；SRE：Supervision + Resistance Exercise

**Supplementary Figure S4 Paired meta-sensitivity analysis of 6MWD.**

**Supplementary Figure S5: Results of pairwise comparisons between groups for the PWV.**

Note：CT：Conventional Treatment；AE：Aerobic Exercise；IHE：Isometric Handgrip Exercise；AERT：Aerobic Exercise + Resistance Training；QD：Qigong + Dancing；TCC：Tai Chi Chuan；SE：Stretching Exercise

**Supplementary Figure S6: Pooled Forest Plot for PWV scores, AE and CT, AERT and CT, IHE and CT, QD and CT, as well as TCC and CT.**

Note：AE：Aerobic Exercise；AERT：Aerobic Exercise + Resistance Training；IHE：Isometric Handgrip Exercise；QD：Qigong + Dancing；TCC：Tai Chichaun；

**Supplementary Figure S7: Paired meta-sensitivity analysis of PWV.**

**Supplementary Figure S8: Results of pairwise comparisons between groups for the TC.**

Note：CT：Conventional Treatment；AE：Aerobic Exercise；RT：Resistance Training；AERT：Aerobic Exercise + Resistance Training；QD：Qigong + Dancing；TCC：Tai Chi Chuan；BFRT：Resistance Exercise + Blood Flow Restriction；SE：Stretching Exercise

**Supplementary Figure S9: Pooled Forest Plot for TC scores, AE and CT, AERT and CT, BFRT and CT, QD and CT, RT and CT, as well as TCC and CT.**

Note：AE：Aerobic Exercise；RT：Resistance Training；AERT：Aerobic Exercise + Resistance Training；QD：Qigong + Dancing；TCC：Tai Chi Chuan；BFRT：Resistance Exercise + Blood Flow Restriction

**Supplementary Figure S10: Paired meta-sensitivity analysis of TC.**

**Supplementary Figure S11: Results of pairwise comparisons between groups for the TG.**

Note：CT：Conventional Treatment；AE：Aerobic Exercise；RT：Resistance Training；QD：Qigong + Dancing；TCC：Tai Chi Chuan；BFRT：Resistance Exercise + Blood Flow Restriction

**Supplementary Figure S12: Pooled Forest Plot for TG scores, AE and CT, BFRT and CT, QD and CT, RT and CT,as well as TCC and CT.**

Note：AE：Aerobic Exercise；RT：Resistance Training；QD：Qigong + Dancing；TCC：Tai Chi Chuan；BFRT：Resistance Exercise + Blood Flow Restriction

**Supplementary Figure S13: Paired meta-sensitivity analysis of TG.**

**Supplementary Figure S14: Results of pairwise comparisons between groups for the HDL-C.**

Note：CT：Conventional Treatment；AE：Aerobic Exercise；RT：Resistance Training；AERT：Aerobic Exercise + Resistance Training；QD：Qigong + Dancing；TCC：Tai Chi Chuan；

**Supplementary Figure S15: Pooled Forest Plot for HDL-C scores, AE and CT, AERT and CT, QD and CT, RT and CT,as well as TCC and CT.**

Note：AE：Aerobic Exercise；RT：Resistance Training；AERT：Aerobic Exercise + Resistance Training；TCC：Tai Chi Chuan；QD：Qigong + Dancing

**Supplementary Figure S16: Paired meta-sensitivity analysis of HDL-C.**

**Supplementary Figure S17: Results of pairwise comparisons between groups for the LDL-C.**

Note：CT：Conventional Treatment；AE：Aerobic Exercise；RT：Resistance Training；AERT：Aerobic Exercise + Resistance Training；TCC：Tai Chi Chuan;BFRT：Resistance Exercise + Blood Flow Restriction

**Supplementary Figure S18:** **Pooled Forest Plot for LDL-C scores, AE and CT, AERT and CT, BFRT and CT, RT and CT,as well as TCC and CT.**

Note：AE：Aerobic Exercise；RT：Resistance Training；AERT：Aerobic Exercise + Resistance Training；TCC：Tai Chi Chuan;BFRT：Resistance Exercise + Blood Flow Restriction

**Supplementary Figure S19: Paired meta-sensitivity analysis of LDL-C.**
